# Discovery of Triterpenoids as Reversible Inhibitors of α/β-hydrolase Domain Containing 12 (ABHD12)

**DOI:** 10.1371/journal.pone.0098286

**Published:** 2014-05-30

**Authors:** Teija Parkkari, Raisa Haavikko, Tuomo Laitinen, Dina Navia-Paldanius, Roosa Rytilahti, Miia Vaara, Marko Lehtonen, Sami Alakurtti, Jari Yli-Kauhaluoma, Tapio Nevalainen, Juha R. Savinainen, Jarmo T. Laitinen

**Affiliations:** 1 School of Medicine, Institute of Biomedicine, University of Eastern Finland, Kuopio, Finland; 2 School of Pharmacy, Faculty of Health Sciences, University of Eastern Finland, Kuopio, Finland; 3 Division of Pharmaceutical Chemistry and Technology, Faculty of Pharmacy, University of Helsinki, Helsinki, Finland; 4 VTT Technical Research Centre of Finland, Helsinki, Finland; Stanford University, United States of America

## Abstract

**Background:**

α/β-hydrolase domain containing (ABHD)12 is a recently discovered serine hydrolase that acts *in vivo* as a lysophospholipase for lysophosphatidylserine. Dysfunctional ABHD12 has been linked to the rare neurodegenerative disorder called PHARC (polyneuropathy, hearing loss, ataxia, retinosis pigmentosa, cataract). *In vitro*, ABHD12 has been implicated in the metabolism of the endocannabinoid 2-arachidonoylglycerol (2-AG). Further studies on ABHD12 function are hampered as no selective inhibitor have been identified to date. In contrast to the situation with the other endocannabinoid hydrolases, ABHD12 has remained a challenging target for inhibitor development as no crystal structures are available to facilitate drug design.

**Methodology/Principal Findings:**

Here we report the unexpected discovery that certain triterpene-based structures inhibit human ABHD12 hydrolase activity in a reversible manner, the best compounds showing submicromolar potency. Based on structure activity relationship (SAR) data collected for 68 natural and synthetic triterpenoid structures, a pharmacophore model has been constructed. A pentacyclic triterpene backbone with carboxyl group at position 17, small hydrophobic substituent at the position 4, hydrogen bond donor or acceptor at position 3 accompanied with four axial methyl substituents was found crucial for ABHD12 inhibitor activity. Although the triterpenoids typically may have multiple protein targets, we witnessed unprecedented selectivity for ABHD12 among the metabolic serine hydrolases, as activity-based protein profiling of mouse brain membrane proteome indicated that the representative ABHD12 inhibitors did not inhibit other serine hydrolases, nor did they target cannabinoid receptors.

**Conclusions/Significance:**

We have identified reversibly-acting triterpene-based inhibitors that show remarkable selectivity for ABHD12 over other metabolic serine hydrolases. Based on SAR data, we have constructed the first pharmacophore model of ABHD12 inhibitors. This model should pave the way for further discovery of novel lead structures for ABHD12 selective inhibitors.

## Introduction

The serine hydrolase α/β-hydrolase domain containing (ABHD)12 is a membrane-bound enzyme that together with monoacylglycerol lipase (MAGL) and ABHD6 contributes to the metabolism of the endocannabinoid 2-arachidonoylglycerol (2-AG) *in vitro* [1]. *In vivo*, ABHD12 serves as a lysophospholipase showing preference towards lysophosphatidylserine (LPS) in the mammalian nervous system [2]. Even though ABHD12 is still poorly characterized, recently developed ABHD12^−/−^ mice have shed some light to its possible physiological functions. In the study of Blankman et al. (2013), ABHD12 deficient mice developed age-dependent symptoms that resemble the human neurodegenerative disorder PHARC (polyneuropathy, hearing loss, ataxia, retinosis pigmentosa, cataract). Authors suggested that the disrupted LPS metabolism and resulting neuroinflammation may form one of the molecular basis for PHARC [3].

Tissue distribution and subcellular localization of MAGL, ABHD6 and ABHD12 are different, suggesting that these hydrolases could control different pools of 2-AG [1]. An active site of ABHD12 is predicted to face the lumen and/or extracellular space and in the latter position ABHD12 could possibly metabolize extracellular pool of 2-AG. We have recently delineated the monoacylglycerol (MAG) substrate preferences of ABHD12 *in vitro* and found that unlike MAGL, ABHD12 (and ABHD6) prefers the 1(3)-isomers of unsaturated MAGs over the 2-isomers [4]. More detailed pharmacological studies with ABHD12 have been limited due to the lack of selective inhibitor(s). Preliminary inhibitor profiling has shown that the universal lipase/serine hydrolase inhibitors tetrahydrolipstatin (THL, Figure S1) and methyl arachidonyl fluorophosphonate (MAFP, Figure S1) relatively potently inhibit ABHD12 [4]. ABHD12 has remained a challenging target for inhibitor development as there are no crystal structures available, number of known inhibitors is low and the existing activity data are limited.

In order to find novel lead structures for selective inhibitors of recently discovered serine hydrolases, exploring the activity of natural compounds may offer valuable information for this developing process. For instance, plant-derived pentacyclic triterpenes such as betulinic, oleanolic and ursolic acid are interesting molecules as they all are bioactive and widespread in nature and their therapeutic potential is well documented [5]–[9] see also reviews [10]–[16] and references cited therein. In addition, their multi-targeting biological activity, low toxicity, easy availability, and core structure offering good starting point for chemical modifications, make triterpenoids appealing source for the drug discovery. Along this line, recent studies have revealed that triterpenes may include potential candidates for novel inhibitors of e.g. endocannabinoid hydrolases. Indeed, pristimerin has been shown to inhibit MAGL activity in *in vitro* studies [17], [18]. In another study, a mixture of α/β-amyrin (ursane and oleanane-type triterpenoids, Figure S2) was shown to reduce inflammatory and neuropathic hyperalgesia in mice through activation of the cannabinoid CB_1_ (CB_1_R) and CB_2_ (CB_2_R) receptors [19]. Interestingly, despite their high affinity towards CB_1_R, the compounds failed to show any cannabimimetic effects in the tetrad test. In addition, α- and β-amyrin were reported to inhibit 2-AG-hydrolysis in pig brain homogenates [20]. The molecular target of this action was not identified.

Our preliminary screening efforts to identify novel serine hydrolase inhibitors among various chemical compounds revealed unexpectedly that ursolic acid was able to selectively inhibit ABHD12 with negligible effect on ABHD6 or MAGL activity. Inspired by this finding, we selected various commercial triterpenes/triterpenoids as well as recently reported betulin-based triterpenes for further evaluation. In this paper, we report the inhibitory activity of these compounds towards human ABHD12. Based on the activity data we have established preliminary structure-activity relationships (SAR) and constructed the first pharmacophore model for betulin-based triterpenes. This model should prove useful in the discovery of novel lead structures for ABHD12 selective inhibitors. Although the triterpenoids typically interact with multiple protein targets, we witnessed unprecedented selectivity towards ABHD12 among the metabolic serine hydrolases, as activity-based protein profiling (ABPP) of mouse brain membrane proteome indicated that the representative ABHD12 inhibitors did not inhibit other serine hydrolases, nor did they target cannabinoid receptors.

## Results and Discussion

### Structure-activity relationship (SAR) studies

Pentacyclic triterpenes can be classified into three different groups: lupanes, oleananes and ursanes. Derivatives of triterpenes are called triterpenoids. In this study, commercially available triterpenes **1–11** and triterpenoids **12–15** were purchased from different chemical vendors and tested for their ability to inhibit hydrolase activity in lysates of HEK293 cells transiently overexpressing human ABHD12 (hABHD12) [4]. The inhibition data are presented in Table 1 (detailed chemical structures of the tested compounds are presented in Figure S2). In the lupane series (**1–3**), an importance of a carboxyl group at position 17 was shown as betulinic acid (**1**) had the highest inhibitory activity (IC_50_ = 2.5 µM). However, lipophilicity differences should also be taken into consideration as the compound with the lowest logD (Table 1) also had the highest inhibitory activity. In the ursane series (**4–6**), similar effect of the carboxyl group at position 17 was observed as ursolic acid (**4**) showed higher inhibition activity compared to α-amyrin (**6**) that has a methyl group at this position. Asiatic acid (**5**), which has a primary hydroxyl group at the position 4, was completely devoid of activity, demonstrating the importance of this position for hABHD12 inhibition. Notably, asiatic acid had the highest water solubility of the entire series which, in this case, did not lead to higher activity. Asiatic acid also has an extra hydroxyl group at position 2. However, it can be concluded that this hydroxyl group was actually favored as maslinic acid (**8**) belonging to the oleanane series, had the same substitution and this feature greatly improved the inhibitory activity. In fact, among the 15 commercial compounds tested, maslinic acid was the best hABHD12 inhibitor having an IC_50_ value of 1.3 µM. The oleanane series (**7–11**) further confirmed our findings that dimethyl at position 4 in combination with a carboxyl group at position 17 were important features for hABHD12 inhibition. Finally, we tested four triterpenoids, 2-cyano-3,12-dioxo-oleana-1,9(11)-dien-28-oic acid (CDDO) (**12**), CDDO methyl ester (**13**), celastrol (**14**), and the established MAGL inhibitor pristimerin (**15**). All four derivatives failed to show any inhibition of hABHD12 and the findings with pristimerin are in agreement with those in the study by King et al. [17] where pristimerin was tested against different endocannabinoid targets. Poor inhibitory activity of triterpenoids **12–15** allowed us to conclude that triterpene backbone was crucial for the hABHD12 inhibitor activity.

**Table 1:** Inhibitor potency in hABHD12-HEK293 lysates for commercially available triterpenes (**1–11**) and triterpenoids (**12–15**) as well as their calculated lipophilicity values (logD).

Compound	General name	Remaining activity at 10 µM % ± *s.e.m.*	-logIC_50_±*s.e.m.* [IC_50_] (max inhibition)	logD^1^ (pH 7.4)
**1**	Betulinic acid		5.60±0.18^2^ [2.5 µM]	4.04
**2**	Betulin	∼75		6.17
**3**	Lupeol	NI		7.45
**4**	Ursolic acid	∼30	5.74±0.14^2^ [1.8 µM]	3.98
**5**	Asiatic acid	NI		1.62
**6**	α-Amyrin	NI		7.39
**7**	Oleanolic acid	∼20	5.80±0.07^2^ [1.6 µM]	3.99
**8**	Maslinic acid		5.90±0.04 [1.3 µM] (89%)	2.92
**9**	Hederagenin	78±3.0		2.71
**10**	β-Amyrin	NI		7.40
**11**	α-Boswellic acid	NI		3.84
**12**	CDDO	NI		3.68
**13**	CDDO methyl ester	NI		6.55
**14**	Celastrol	NI		2.75
**15**	Pristimerin	NI		5.47

1 logD values have been calculated using ChemAxon Marvin 6.0 software.

2 Valueas are -logIC_50_±*s.d.*, *n* = 2.

NI no inhibition.

Data are mean ± SEM from three independent experiments.

**Figure 1:**
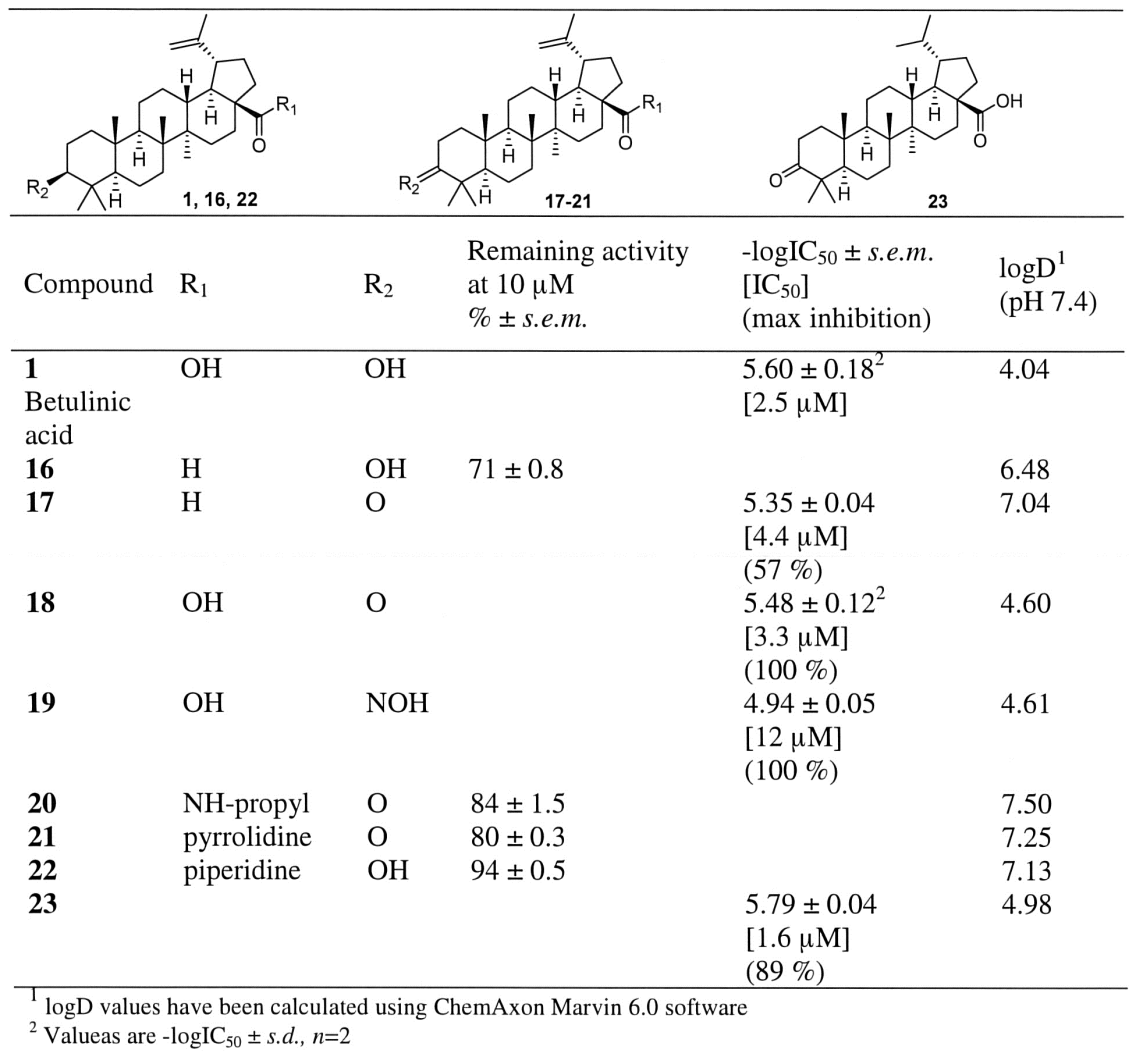
Inhibitor potency in hABHD12-HEK293 lysates for compounds 16–23 as well as their calculated lipophilicity values (logD). Data are mean ± SEM from three independent experiments.

**Figure 2:**
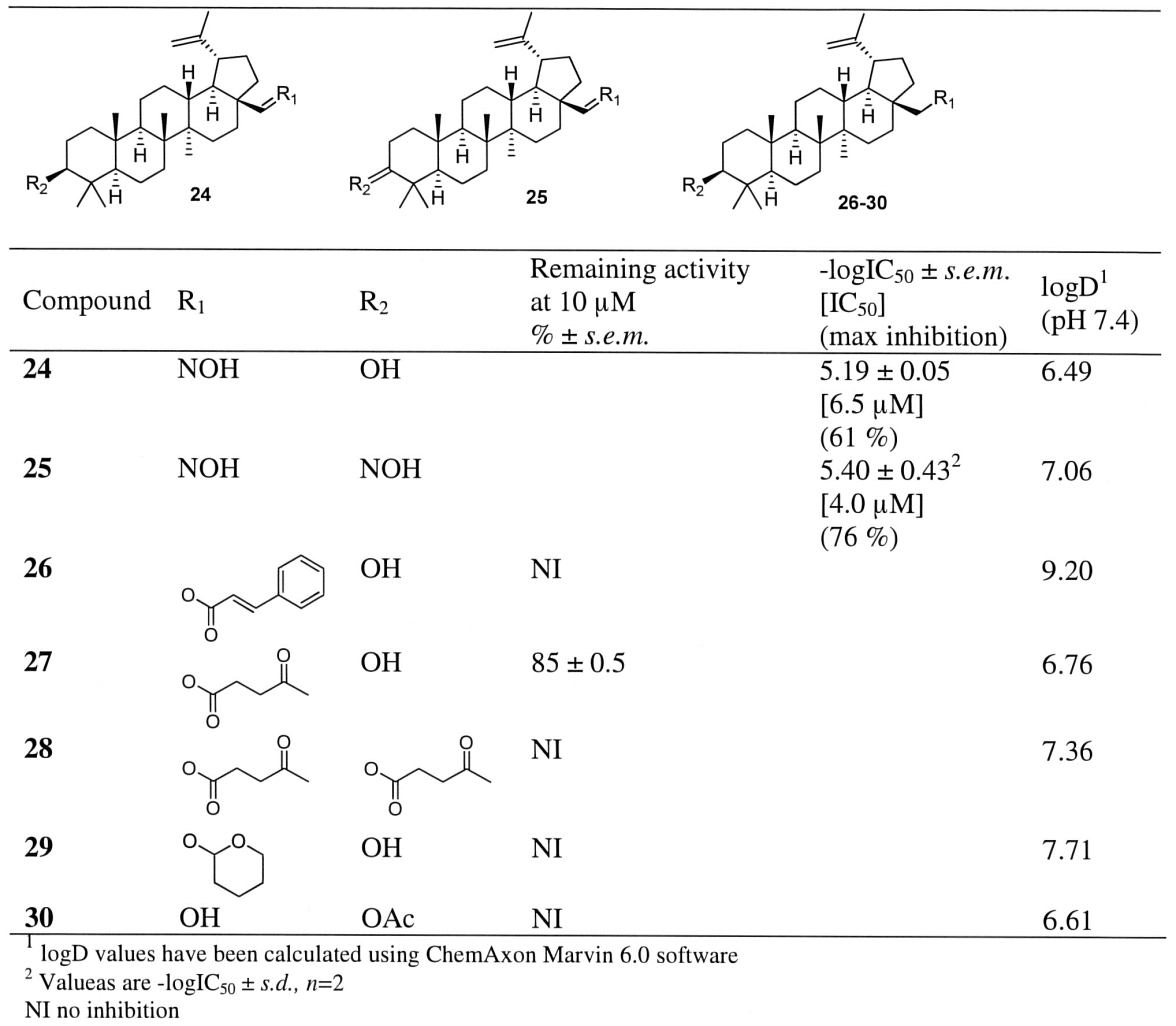
Inhibitor potency in hABHD12-HEK293 lysates for compounds 24–30 as well as their calculated lipophilicity values (logD). Data are mean ± SEM from three independent experiments.

**Figure 3:**
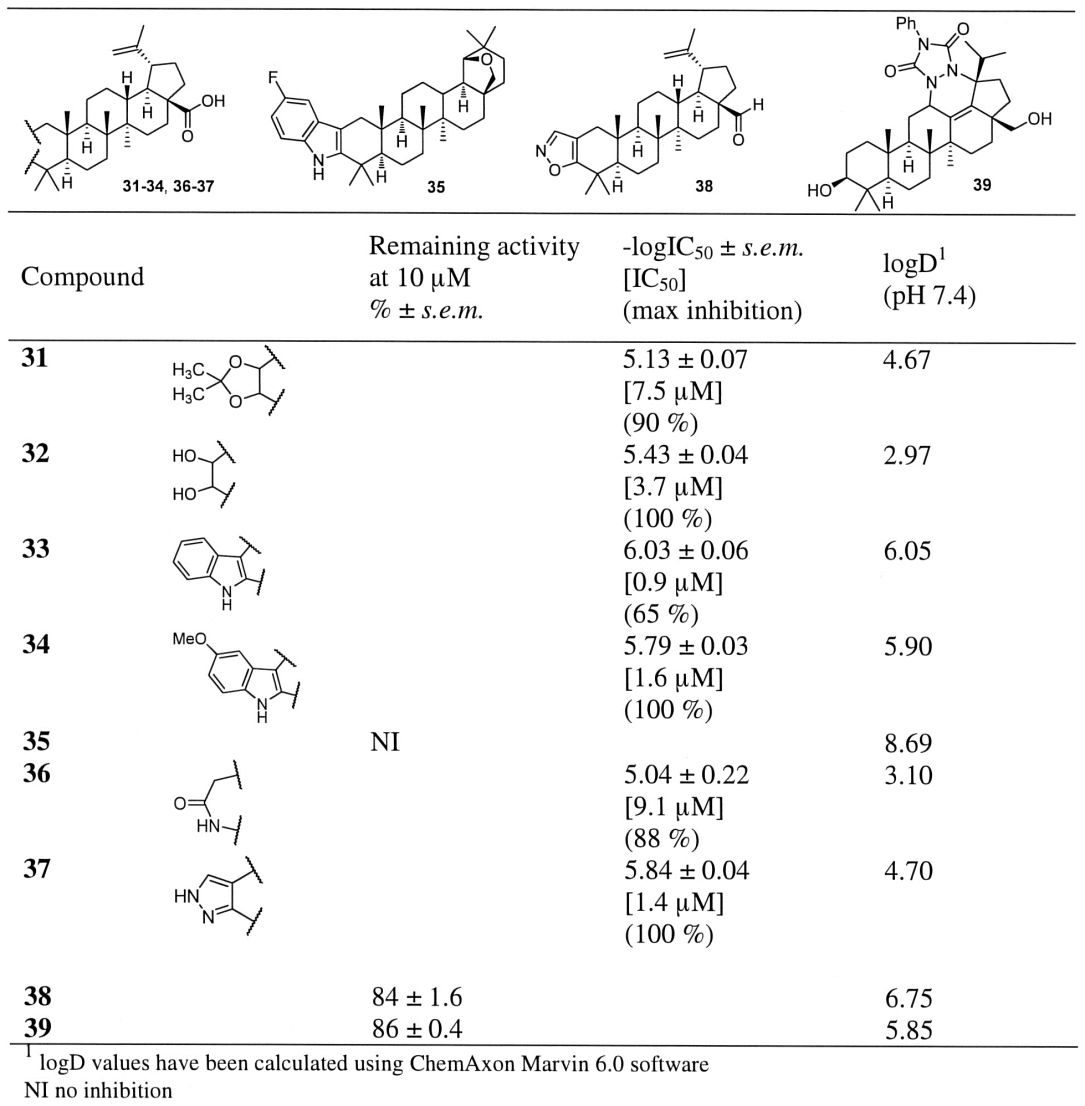
Inhibitor potency in hABHD12-HEK293 lysates for compounds 31–39 as well as their calculated lipophilicity values (logD). Data are mean ± SEM from three independent experiments.

**Figure 4:**
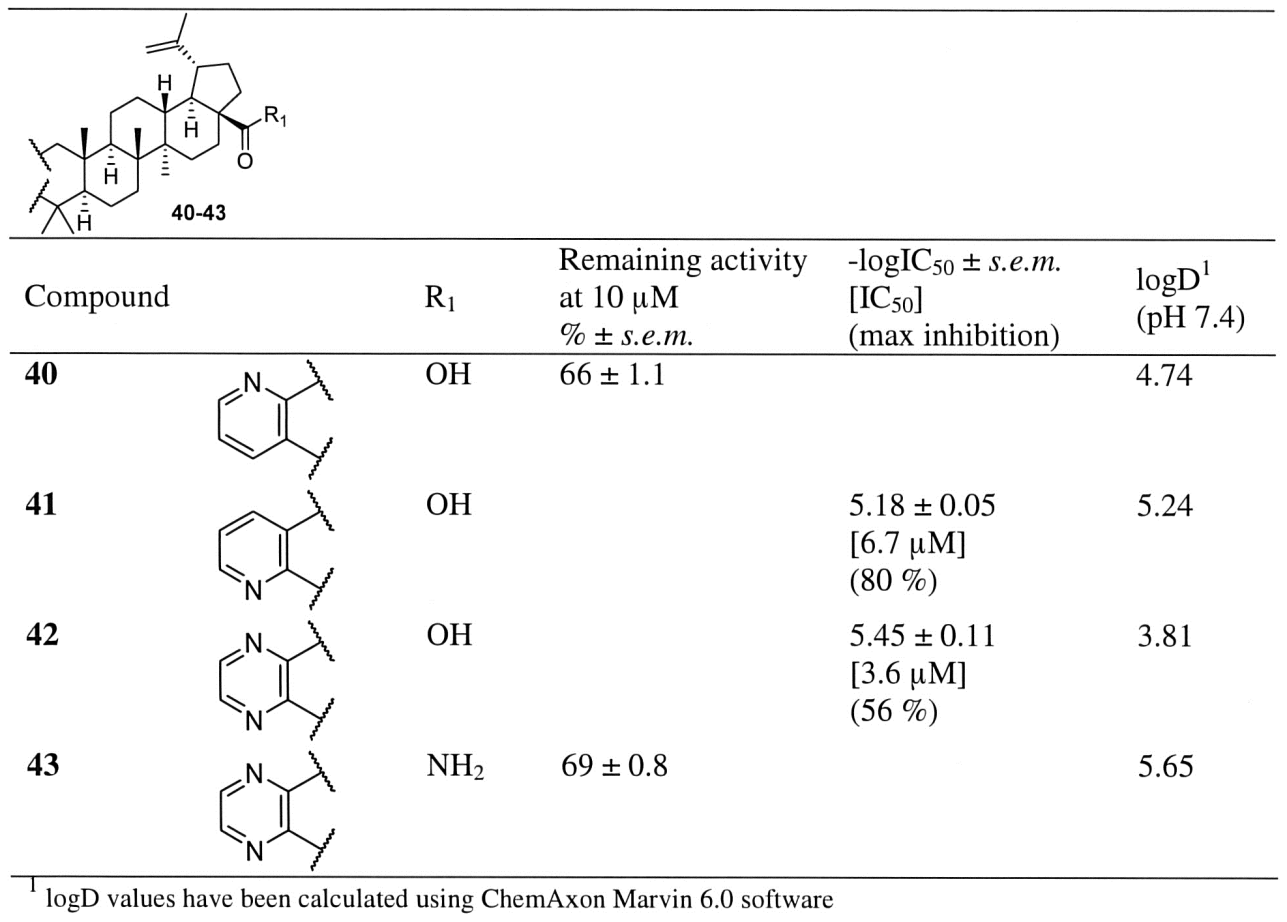
Inhibitor potency in hABHD12-HEK293 lysates for compounds 40–43 as well as their calculated lipophilicity values (logD). Data are mean ± SEM from three independent experiments.

As betulinic acid (**1**), ursolic acid (**4**) and oleanolic acid (**7**) had only minor differences in their inhibitory activities, neither the size of the ring E (Figure S1) nor its substituents have a role in hABHD12 inhibition. In order to establish additional structural features that are critical for hABHD12 inhibition, we chose a series of previously reported derivatives of betulinic acid (**16–43** Table 1 and Figures 1–4, for compounds **44–68** see Table S3) for further evaluation. Importance of the carboxyl group at position 17 was further verified by testing an aldehyde **16** which only weakly inhibited hABHD12 (∼30%) at 10 µM concentration (Figure 1). When comparing two similar aldehydes (**16** and **17**), the inhibition was enhanced to moderate level when hydroxyl substituent at position 3 was replaced to carbonyl, i.e. a plain hydrogen bond accepting group. An amide bond (**20–22**, Figure 1) as well as an insertion of an ester (**26–28**, Figure 2) or ether (**29**, Figure 2) similarly decreased inhibitor activity. When carboxyl group was replaced with an oximino group (**24**, Figure 2), modest inhibitory activity was observed (IC_50_ 6.5 µM). Inhibitory activity of the oxime **24** was retained by replacing hydroxyl group at position 3 with another oximino group (**25**, Figure 2). When carboxyl group at position 17 was retained and an oximino group was added at position 3 (**19**, Figure 1), decreased inhibitory activity was observed. However, it was interesting that compound **19** was able to fully inhibit the enzyme whereas maximum inhibition of the compound **24** was only 61%.

The effect of the modifications on the ring A on hABHD12 inhibitor activity are presented in the Figures 3–4 and Table S3. As shown in the case of maslinic acid, an additional hydroxyl group at the position 2 resulted in good inhibition. We synthesized the corresponding betulinic acid derivative **32** (Figure 3) and observed that the activity of this compound was similar to that of the parent betulinic acid (**1**). Additional heterocyclic ring system attached to the ring A generally gave good inhibition. For example, when hydroxyl groups at positions 2 and 3 were protected as an acetonide (**31**), modest inhibitory activity (IC_50_ 7.5 µM) was observed. Replacement of a ring A with a lactam ring (**36**) resulted in modest inhibitory activity (IC_50_ 9.1 µM), however, the lactam ring also decreased selectivity, as compound **35** also inhibited MAGL (∼50% inhibition at 10 µM concentration, Table S1). Introduction of a pyridine or a pyrazine ring (**40–43**, Figure 4) or an indole ring (**33–34**, Figure 3) revealed an important structural feature. The position of a nitrogen atom in the pyridine ring turned out to be important for the inhibitory activity as the compound **41** showed improved activity over the compound **40**. Activity was further improved by replacing the pyridine ring with an indole ring (**33–34**, Figure 3) or a pyrazole (**37**, Figure 3). In fact, compound **33** was the most potent compound of the entire series having an IC_50_ value of 0.9 µM (maximum inhibition 65%). As evidenced by the total loss of the inhibitory activity in the case of the indole-fused allobetulin derivative **35** (Figure 3), the carboxyl group at position 17 was still needed for inhibitory activity. However, one should also keep in mind that the solubility of allobetulin derivative was very low. Finally, poor inhibitory activity of the isoxazole **38** allowed us to conclude that a functional group with a hydrogen bond donor or acceptor at carbon 3 was crucial for ABHD12 inhibitory activity.

In summary, the above-described SAR studies allowed us to identify four key determinants for hABHD12 inhibition potency and efficacy. These key features played an important role in building a pharmacophore model of ABHD12 that is described later in this chapter. (i) Shape complementarity of the triterpene skeleton accompanied with four axial methyl substituents likely play an important role in inhibitor binding. Additional double bonds within the skeleton (CDDO and celastrol) affect the overall planar shape of triterpene scaffold, leading to total lack of inhibitory activity. (ii) Carboxyl group at position 17 in triterpene core structure is of crucial importance, as basically any modification at this position reduced, or fully eliminated inhibitory activity. It is known from previous studies that the carboxyl group at this position is crucial also in many other biological targets [21]–[23]. (iii) Small, hydrophobic substituents at the position 4 are required, as asiatic acid (**5**) and hederagenin (**9**) did not inhibit hABHD12. (iv) As summarized in Figure S3, hydrogen bond donor or acceptor attached to position 3 was a key feature required for high inhibitory potency. This was further illustrated by pyridine and pyrazine derivatives **40–42**. Compounds **41** and **42** have a nitrogen at this position and show moderate inhibitory activity (IC_50_ values 6.7 and 3.6 µM, respectively). In contrast, no atom capable of hydrogen bonding is present in the compound **40**, causing dramatic decrease of inhibitory activity (34% inhibition at 10 µM). A good pair of compounds for comparison was **37** and **38**. The pyrazole derivative **37** was one of the most potent compounds in the series. On the other hand, when an aromatic nitrogen was replaced by an oxygen (isoxazole **38**), a complete loss of the inhibitory activity was observed. This was due to the fact that oxygen within an aromatic ring cannot form hydrogen bond. The most promising synthetic compounds in the series were compounds **33** and **34** which both have an indole ring attached to ring A, and thus, a nitrogen atom at this crucial hydrogen bonding position. The compound **34** has an electron donating methoxy group at the indole ring which may cause tighter interaction between indole nitrogen and amino acid residues of the enzyme. In addition, good inhibitory activity of this methoxy derivative also implies that there is additional space for bulkier substituents in this direction. Strength of a hydrogen bond may also explain why these compounds were equally potent in inhibiting hABHD12 activity but their maximal inhibition was significantly different (65% and 100%, respectively). Similar trend in efficacy was observed with the compounds **41** and **42**, i.e. additional nitrogen in pyrazine decreased efficacy (maximal inhibition 80 and 56%, respectively). Collectively, the above data demonstrate the importance of a hydrogen bond donor at position 3. However, as betulinic acid (**1**) that can act both as a hydrogen bond acceptor and a donor, and compound **18** that is a hydrogen bond acceptor, both showed good inhibitory activity, we were able to conclude that both hydrogen bond donor and acceptor are tolerated at this position.

The key finding that both hydrogen bond donor and acceptor at position 3 are able to form good interactions with the enzyme led us to hypothesize that these interactions might involve a serine residue, possibly the catalytic serine (S246) of ABHD12, previously identified by site-directed mutagenesis [4]. However, due to the reversible nature of triterpenoid inhibition (see below), this hypothesis could not be experimentally tested.

### Triterpenoids inhibit hABHD12 in a reversible manner

There are no suitable functional groups in the triterpenoid scaffold that could irreversibly react with catalytic residues of the serine hydrolases. This has been previously shown with pristimerin that inhibits MAGL in a reversible manner [17]. To test whether the triterpenoids also reversibly inhibit hABHD12, we assessed time-dependency of inhibitor potency following rapid, 40-fold dilution of the enzyme-inhibitor complex. We determined the IC_50_ values for selected triterpenoids from kinetically recorded data at time points 10, 20, 30, 60 and 90 minutes. A statistically significant, time-dependent drop in inhibitor potency was evident for the tested triterpenoids, indicating rapid dissociation of the inhibitor from the enzyme's active site (Figure S4). In contrast, the IC_50_ values for the reference compound THL remained constant regardless of the incubation time, indicating that within the time-frame studied, the β-lactone irreversibly inhibited hABHD12.

### A ligand-based pharmacophore model for ABHD12

**Figure 5:**
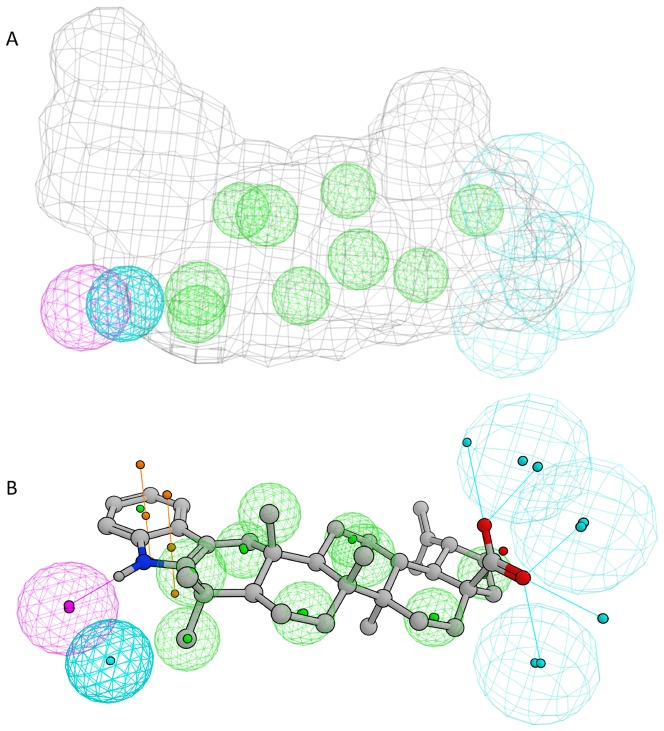
Collection of the features used to define the pharmacophoric model for the binding of the triterpenes to the ABHD12. Color coding of pharmacophoric features: green, hydrophobic site; magenta, acceptor site; cyan, donor site; gray cage, ligand shape constraint. **B**. The best solution for the alignment of the most active compound (**33**) to pharmacophore model (the same color coding as above except the ligand surface omitted for the clarity).

Low structural homology to experimental template structures prevented a creation of a comparative model of ABHD12 that could have been used in docking studies. Before looking at the SAR in more detail, we wished to clarify whether the chemical structures of the *in vivo* substrate LPS, the irreversible inhibitor THL, and the triterpene inhibitor betulinic acid could be aligned. Interestingly, molecular superimposition disclosed that there were striking similarities, not only in the topology and dimensions of the three compounds, but also in the distance and orientation of the functional groups (Figure S5). Encouraged by these findings, we proceeded to the collected SAR data that included a total of 68 compounds of which 18 are classified as active and 50 as inactive. Based on these compounds, we constructed a model that consists of 14 pharmacophoric features (Figure 5). When a partial match of 11/14 was used, all 18 active triterpenoids satisfied the query and only 4 false positives were found (false positive compounds: betulin, asiatic acid, hederagenin and compound **16**, false positive rate  = 0.08). It can be concluded that some inactive compounds were structurally so close to the active compounds that they cannot be easily ruled out by the means of common pharmacophoric criteria.

### Triterpenoids exhibit unprecedented selectivity towards ABHD12 among the serine hydrolases

**Figure 6:**
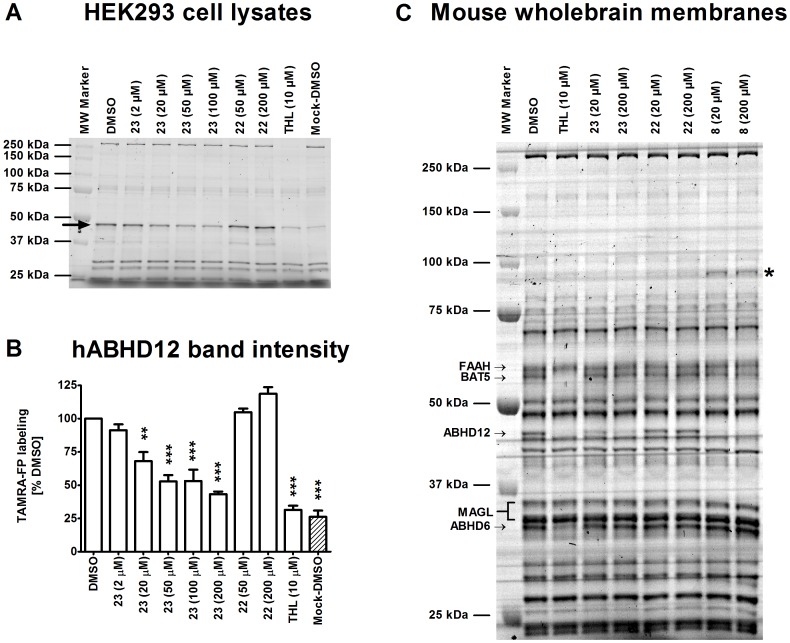
Competitive ABPP to unveil triterpenoid targets among the serine hydrolases in proteomes of HEK293 cell lysates (A–B) and mouse brain membranes (C). Lysates (25 µg) or membranes (100 µg) were treated for 1 h with DMSO or the indicated concentrations of the inhibitors, after which TAMRA-FP labelling was conducted for 5 min (lysates) or for 1 hour (membranes), as described in Methods. The proteins were resolved in 10% SDS-PAGE together with protein standards. TAMRA-FP labeling was visualized after in-gel fluorescence imaging as described in experimental procedures. Molecular weight markers are shown in both gel images. **A**. Transient expression of hABHD12 in HEK293 cells results in the appearance of a ∼46 kDa protein band (black arrow), comigrating together with an endogenous serine hydrolase with similar size. The endogenous band is weakly visible in Mock lysates and does not correspond to ABHD12 [4]. Note that probe labeling to hABHD12 is fully prevented by THL (10 µM), it is also dose-dependently inhibited by compound **23** whereas compound **22** is ineffective. **B**. Quantitative data (mean ± SEM) on the effects of selected triterpenoids on probe labeling of the hABHD12 band (black arrow in A) combined from three independent experiments with hABHD12-HEK lysates. Statistical differences in band intensities were tested using one-way ANOVA, followed by Tukey's multiple comparison test (**P<0.01 and ***P<0.001). **C**. ABHD12 is the sole triterpenoid target among the serine hydrolases in mouse brain membrane proteome. THL (10 µM) was used as a positive control and consistent with previous data [1], [34], [35], THL prevented TAMRA-FP labeling of serine hydrolases migrating at ∼63 kDa (BAT5), ∼46 kDa (ABHD12) and ∼33 kDa (ABHD6). Note that compound **23** and maslinic acid (compound **8**) dose-dependently inhibit TAMRA-FP labeling of ABHD12 already at 20 µM whereas compound **22** is ineffective, even at 200 µM concentration. Note also that with the exception of heightened TAMRA-FP labeling of an unidentified serine hydrolase migrating at ∼90 kDa (indicated by the asterix), no additional targets are evident for maslinic acid or compound **23** among the metabolic serine hydrolases. The gel is representative from three independent ABPP runs with similar outcome.

Triterpenoids possess a “universal” core structure that can be recognized by many proteins. Therefore, the triterpenoids reported in this paper are likely to have additional targets besides ABHD12. Despite this, we were surprised to learn that the representative inhibitors in the triterpenoid series did not hit other targets of the endocannabinoid system including ABHD6, MAGL, fatty acid amide hydrolase (FAAH) or the cannabinoid receptors (Tables S1 and S2). We used activity-based protein profiling (ABPP) with the catalytic serine-targeting probe TAMRA-FP to unveil triterpenoid targets among the metabolic serine hydrolases in lysates of hABHD12-HEK293 cells (Figure 6A–B). These experiments revealed that the active triterpenoid **23** dose-dependently inhibited probe labeling of hABHD12 whereas compound **22** which was inactive in glycerol-based hydrolase assays, lacked activity also in the ABPP assay. We extended the ABPP studies to cover mouse brain membrane proteome and found that compound **23** and maslinic acid (**8**) (but not the inactive compound **22**) selectively inhibited probe labeling of ABHD12 in the brain proteome (Figure 6C). It is noteworthy that with the exception of maslinic acid interfering with probe labeling of an uncharacterized ∼90 kDa protein, no additional targets were evident for the triterpenoids among the brain membrane serine hydrolases (Figure 6C). Thus, although the *in vitro* inhibitor and substrate profiles of ABHD12 were previously shown to partly overlap with those of ABHD6 and MAGL [4], ABHD12 appears to be rather unique among these hydrolases, and more broadly so also among the metabolic serine hydrolases, as it was the sole serine hydrolase targeted by the triterpenoids in native brain membrane proteome.

In conclusion, we report the discovery of the first phytocompounds and their synthetic analogues that inhibit human and mouse ABHD12. The studied compounds belong to the class of triterpenoids that are known to possess wide-ranging therapeutic effects. The best compounds fully inhibited hABHD12 with the IC_50_ values in the submicromolar range. Further mechanistic studies using representative compounds, including maslinic acid, showed that ABHD12 inhibition was reversible. The compounds did not inhibit the endocannabinoid hydrolases such as ABHD6, MAGL and FAAH, nor did they show appreciable activity towards the cannabinoid receptors. Activity-based protein profiling of mouse brain membrane proteome with a serine hydrolase-targeting probe revealed that the triterpenoids selectively inhibited ABHD12 with no additional targets evident among the metabolic serine hydrolases. Thus contrary to preconceived thinking, the triterpenoids showed unprecedented selectivity for ABHD12, not only over other serine hydrolases but also over cannabinoid receptors. Finally, using our SAR analysis with the presently described betulin-based compound series, we have disclosed important structural features required for ABHD12 inhibition. We have used these data in the development of the first pharmacophore model for ABHD12. This model should be useful in further studies aiming at the discovery of novel lead structures for ABHD12 inhibitors.

## Methods

### Chemistry

Compounds **1**, **3–7**, **9–11** and **14–15** were purchased from Sigma-Aldrich and compounds **2**, **8** and **12–13** from Cayman Chemical Company. Commercially available compounds were used without further purification or structural verification. Synthesis procedures and structural verification of the compounds **16–43** (with the exception of compound **32** and **40**) have been previously reported [24]–[27]. Structures of inactive compound **44–68** have been presented in Table S3. Synthesis procedures and structural verification have been previously reported (with the exception of the compound **68**) [24]–[27].

### Synthesis procedures (compounds 32, 40, 68)

#### 2*β*,3*β*-Dihydroxylup-20(29)-en-28-oic acid (32)

A mixture of betulonic acid (68 mg, 0.15 mmol) and potassium *tert*-butoxide (604 mg, 5.40 mmol) in *tert*-butanol (7.0 mL) was stirred with constant air flow at 40°C for 2 h. Then 1 M hydrochloric acid was added and the resulting mixture was extracted with EtOAc several times. The combined organic phases were washed with water and brine, dried over anhydrous Na_2_SO_4_ and evaporated to give a white crystalline solid 2-hydroxy-3-oxolupa-1(2),20(29)-dien-28-oic acid (69 mg, 98%). ^1^H NMR (300 MHz, CDCl_3_) *δ* 6.44 (s, 1H), 4.75 (s, 1H), 4.63 (s, 1H), 3.11–2.93 (m, 1H), 2.38–2.15 (m, 2H), 1.70 (s, 3H), 1.52 (m, 15H), 1.20 (s, 3H), 1.12 (s, 3H), 1.10 (s, 3H), 1.01 (s, 3H), 0.98 (s, 3H). A solution of 2-hydroxy-3-oxolupa-1(2),20(29)-dien-28-oic acid (69 mg, 0.15 mmol) in tetrahydrofuran (3 mL) and ethanol (1 mL) was kept on ice bath and NaBH_4_ (18 mg, 0.48 mmol) was added to the solution. The resulting mixture was stirred at room temperature for 2 h. Then 1 M hydrochloric acid was added to the reaction mixture, and it was extracted with EtOAc, washed with water and brine, dried over anhydrous Na_2_SO_4_, and evaporated. The crude product was purified with SiO_2_ column chromatography (10–100% EtOAc/*n*-hexane) to yield 2*β*,3*β*-dihydroxylup-20(29)-en-28-oic acid as a white crystalline solid (25 mg, 35%). ^1^H NMR (300 MHz, (CD_3_)_2_CO) *δ* 4.73 (m, 1H), 4.60 (m, 1H), 4.01 (m, 1H), 3.49 (br s, 1H), 3.13 (d, *J* = 4.0 Hz, 1H), 3.06 (m, 1H), 2.32 (m, 2H), 2.13 (dd, *J* = 14.2, 2.9 Hz, 1H), 1.94 (m, 2H), 1.70 (s, 3H), 1.57–1.36 (m, 9H), 1.19 (s, 3H), 1.02 (s, 3H), 0.98 (s, 6H), 0.97 (s, 3H); ^13^C NMR (75 MHz, (CD_3_)_2_CO) *δ* 177.7, 151.8, 110.2, 78.8, 72.0, 57.0, 56.5, 52.0, 50.2, 48.2, 45.6, 43.6, 41.9, 39.3, 39.2, 38.0, 37.8, 35.4, 33.1, 31.6, 30.6, 26.7, 22.1, 19.7, 19.1, 17.9, 17.8, 16.8, 15.3. NMR spectral data is consistent with those previously reported [28].

#### Lupa-2,20(29)-dieno[2,3-*b*]pyridin-28-oic acid. (i) Benzyl betulonate (40)

To a mixture of betulonic acid (0.500 g, 1.10 mmol) and K_2_CO_3_ (304 mg, 2.20 mmol) in DMF (10 mL) at 55°C, benzyl bromide (244 mg, 1.43 mmol) was added dropwise. After stirring the reaction mixture at 55°C overnight, water was added, and the resulting solution was extracted with EtOAc. The organic phase was washed with water for several times and subsequently with brine, dried over anhydrous Na_2_SO_4_ and evaporated. The crude product was purified with SiO_2_ column chromatography (20–50% EtOAc/*n*-hexane) to yield benzyl betulonate as a white crystalline solid (388 mg, 65%). ^1^H NMR (300 MHz, CDCl_3_) *δ* 7.35 (m, 5H), 5.12 (q, *J* = 12.3 Hz, 2H), 4.72 (s, 1H), 4.60 (s, 1H), 3.16–2.93 (m, 1H), 2.35 (m, 4H), 1.97–1.79 (m, 3H), 1.66 (s, 3H), 1.49–1.19 (m, 15H), 1.06 (s, 3H), 1.01 (s, 3H), 0.95 (s, 3H), 0.90 (s, 3H), 0.79 (s, 3H). **(ii) Benzyl 2-hydroxy-3-oxolupa-1(2),20(29)-dien-28-oate.** A mixture of benzyl betulonate (822 mg, 1.55 mmol) and potassium *tert*-butoxide (2.38 g, 43.8 mmol) in *tert*-butanol (50 mL) was stirred with constant air flow at 40°C for 2 h. Then 1 M hydrochloric acid was added and the resulting mixture was extracted with EtOAc several times. The combined organic phases were washed with water and brine, dried over anhydrous Na_2_SO_4_ and evaporated to give a white crystalline solid benzyl 2-hydroxy-3-oxolupa-1(2),20(29)-dien-28-oate (817 mg, quant.). ^1^H NMR (300 MHz, CDCl_3_) *δ* 7.43–7.28 (m, 4H), 5.13 (q, *J* = 12.3 Hz, 2H), 4.72 (s, 1H), 4.61 (s, 1H), 3.01 (s, 1H), 2.25 (m, 3H), 1.65 (s, 3H), 1.62–1.31 (m, 14H), 1.18 (s, 3H), 1.09 (s, 6H), 1.18 (s, 3H), 1.09 (s, 3H), 0.96 (s, 3H), 0.79 (s, 3H). **(iii) Benzyl 2** ***α*** **,3** ***β*** **-dihydroxylup-20(29)-en-28-oate.** A solution of benzyl 2-hydroxy-3-oxolupa-1(2),20(29)-dien-28-oate (817 mg, 1.46 mmol) in tetrahydrofuran (20 mL) and ethanol (4 mL) was kept on ice bath and NaBH_4_ (159 mg, 2.88 mmol) was added to the solution. The resulting mixture was stirred at room temperature for 1.5 h. Then 1 M hydrochloric acid was added to the reaction mixture, and it was extracted with EtOAc, washed three times with water, a saturated aqueous solution of NaHCO_3_, water and brine, dried over anhydrous Na_2_SO_4_, and evaporated. The crude product was purified with SiO_2_ column chromatography (10–50% EtOAc/*n*-hexane) to yield benzyl 2*α*,3*β*-dihydroxylup-20(29)-en-28-oate as a white crystalline solid (605 mg, 74%). ^1^H NMR (300 MHz, CDCl_3_) *δ* 7.50–7.26 (m, 5H), 5.12 (q, *J* = 12.3 Hz, 2H), 4.72 (s, 1H), 4.59 (s, 1H), 4.08 (d, *J* = 3.4 Hz, 1H), 3.17 (d, *J* = 4.0 Hz, 1H), 3.02 (d, *J* = 4.6 Hz, 1H), 2.43–2.05 (m, 2H), 1.88 (m, 4H), 1.67 (s, 3H), 1.58–1.18 (m, 14H), 1.17–1.02 (m, 7H), 0.97 (s, 6H), 0.93 (s, 3H), 0.76 (s, 3H). **(iv) A mixture of benzyl 2-oxolup-20(29)-en-28-oate and benzyl 3-oxolup-20(29)-en-28-oate.** A solution of benzyl 2*α*,3*β*-dihydroxylup-20(29)-en-28-oate (605 mg, 1.07 mmol) and *p*-toluenesulfonyl chloride (287 mg, 1.50 mmol) in anhydrous pyridine (10 mL) was stirred at 30°C for 6 h and after that stirring was continued at 60°C for 18 h. The reaction mixture was cooled to room temperature and 1 M hydrochloric acid was added. The resulting mixture was extracted with EtOAc, washed three times with water, a saturated aqueous solution of NaHCO_3_ and brine, dried over anhydrous Na_2_SO_4_ and evaporated. The crude product was purified with SiO_2_ column chromatography (5–20% EtOAc/*n*-hexane) to yield a mixture of two compounds, benzyl 2-oxolup-20(29)-en-28-oate and benzyl 3-oxolup-20(29)-en-28-oate as a white crystalline solid (339 mg, 58%). ^1^H NMR (300 MHz, CDCl_3_) *δ* 7.33 (m, 5H), 5.29–4.95 (m, 2H), 4.72 (s, 1H), 4.59 (s, 1H), 3.17–2.87 (m, 1H), 2.57–2.05 (m, 4H), 2.06–1.79 (m, 3H), 1.68 (s, 3H), 1.54–1.13 (m, 17H), 1.05 (s, 2H), 1.02 (s, 2H), 1.00 (s, 2H), 0.97 (s, 2H), 0.94 (s, 2H), 0.89 (s, 2H), 0.84 (s, 3H), 0.80 (s, 2H), 0.78 (s, 2H), 0.74 (s, 2H). **(v) A mixture of betulonic acid and 2-oxolup-20(29)-en-28-oic acid.** To a solution of benzyl 2-oxolup-20(29)-en-28-oate and benzyl 3-oxolup-20(29)-en-28-oate (330 mg, 0.60 mmol) in tetrahydrofuran (15 mL) was added 10% Pd on carbon (30 mg) under argon. Argon atmosphere was replaced with H_2_, and the resulting mixture was stirred at room temperature for 4.5 h. The reaction mixture was filtered through a thin layer of Celite, and the resulting filtrate was evaporated. The crude product was purified with SiO_2_ column chromatography (10–30% EtOAc/*n*-hexane) to yield a mixture of betulonic acid and 2-oxolup-20(29)-en-28-oic acid as a white crystalline solid (220 mg, 80%). ^1^H NMR (300 MHz, CDCl_3_) *δ* 4.74 (s, 1H), 4.61 (s, 1H), 2.99 (m, 1H), 2.60–2.11 (m, 5H), 2.02–1.78 (m, 3H), 1.72 (s, 4H), 1.57–1.14 (m, 17H), 1.07 (s, 1H), 1.04 (s, 2H), 1.01 (s, 2H), 0.99 (s, 2H), 0.97 (s, 2H), 0.93 (s, 1H), 0.92 (s, 2H), 0.85 (s, 2H), 0.83 (s, 2H). **(vi) Lupa-2,20(29)-dieno[2,3-** ***b*** **]pyridin-28-oic acid.** A mixture of betulonic acid and 2-betulonic acid (220 mg, 0.48 mmol) as well as propargylamine (53 mg, 0.97 mmol) and CuCl_2_ (27 mg, 0.16 mmol) in ethanol (8 mL) was refluxed for 17 h. The resulting solution was filtered and evaporated, and the crude product was purified with SiO_2_ column chromatography (10–50% EtOAc/*n*-hexane) to give lupa-2,20(29)-dieno[2,3-*b*]pyridin-28-oic acid (**35**) as a crystalline product (37 mg, 21%).^1^H NMR (300 MHz, CDCl_3_) *δ* 8.37 (1H, dd, *J* = 4.8, 1.6 Hz, H-31), 7.68–7.61 (1H,m, H-33), 7.13 (1H, dd, *J* = 8.0, 4.8 Hz, H-32), 4.75 (1H, s, H-29), 4.63 (1H, s, H-29), 3.07 (2H, m, H-1), 2.48 (2H, m) 2.31 (2H, m, H-11), 1.98 (2H, m), 1.71 (3H s, H-30), 1.54 (m, 13H), 1.27 (m, 2H), 1.23 (3H, s, H-23/24), 1.18 (3H, s, H-24/23), 1.02 (3H, s, H-27), 1.01 (3H, s, H-26), 0.80 (3H, s, H-25); ^13^C NMR (75 MHz, CDCl_3_) *δ* 180.9 (C-28), 155.0 (C-2), 150.6 (C-20), 146.1 (C-31), 141.0 (C-4), 135.7 (C-33), 121.9 (C-32), 110.0 (C-29), 56.6, 53.0 (C-5), 49.4, 49.0 (C-9), 48.8 (C-1), 47.1 (C-19), 42.7, 40.7 (C-13), 38.7, 37.3, 37.1, 36.8 (C-10), 33.6, 33.2 (C-23/24), 32.4 (C-11), 30.9, 29.9, 25.7 (C-24/23), 21.5, 20.2, 19.7 (C-30), 16.2 (C-25), 15.9 (C-26), 14.9 (C-27); FTIR (ν, cm^−1^): 872, 1132, 1159, 1178, 1449, 1708, 2869, 2943; HRMS: *m/z* calcd for C_33_H_48_NO_2_: 490.3685, found 490.3688 [M+H]^+^.

#### 1-(1*H*-1,2,4-Triazol-1-yl)betulonone (68)

Solution of betulonic acid (0.10 g, 0.22 mmol) and 1,1′-carbonyl-di-(1,2,4-triazole) (216 mg, 1.30 mmol) in tertrahydrofuran was refluxed for 20 h. Solvent was evaporated and the crude product was purified with SiO_2_ column chromatography (10-100% EtOAc/*n*-hexane) to yield 1-(1*H*-1,2,4-triazol-1-yl)betulonone as white solid (55 mg, 49%). ^1^H NMR (300 MHz, CDCl_3_) *δ* 8.91 (s, 1H), 7.98 (s, 1H), 4.76 (s, 1H), 4.64 (s, 1H), 3.08–2.85 (m, 2H), 2.73–2.30 (m, 4H), 1.90 (m, 1H), 1.71 (s, 3H), 1.38 (m, 10H), 1.06 (s, 3H), 1.01 (s, 6H), 0.96 (s, 3H), 0.94 (s, 3H); ^13^C NMR (75 MHz, CDCl_3_) *δ* 218.0, 173.6, 152.4, 150.1, 145.3, 110.2, 58.6, 55.2, 51.2, 50.3, 47.5, 45.8, 42.6, 40.9, 39.8, 37.4, 37.1, 36.4, 34.3, 33.8, 31.7, 30.7, 30.0, 26.8, 25.7, 21.7, 21.2, 19.8, 19.6, 16.2, 16.0, 14.8; FTIR (ν, cm^−1^): 670, 897, 1178, 1271, 1356, 1706, 1736, 2872, 2940; HRMS: *m/z* calcd for C_32_H_47_N_3_NaO_2_: 528.3566, found 528.3563 [M+Na^+^] [29].

### Determination of endocannabinoid hydrolase activity

Glycerol liberated from 1-AG hydrolysis was determined with a sensitive fluorescent assay using lysates of HEK293 cells with transient overexpression of hABHD12, hABHD6 or hMAGL, essentially as previously described [4]. Briefly, following 30 min incubation of DMSO/inhibitor (1 µl) together with hABHD12-HEK293, hABHD6-HEK293 or hMAGL-HEK293 lysates (99 µl, 0.3 µg protein/well) in TEMN-BSA buffer [50 mM Tris-HCl, pH 7.4, 1 mM EDTA, 5 mM MgCl_2_, 100 mM NaCl, 0.5% (w/v) fatty acid-free BSA], 1-AG [12.5 µM final concentration, containing additionally 1% EtOH (v/v)] in glycerol assay mix prepared in TEMN-BSA buffer was added (100 µl) and the incubation continued for 90 min at RT. Fluorescence (λ_ex_ 530; λ_em_ 590 nm) was monitored using a Tecan Infinite M200 plate reader (Tecan Group Ltd., Männedorf, Switzerland). Inhibitory activities of selected compounds towards FAAH were determined using membranes of hFAAH overexpressing COS-7 cells, essentially as previously described [30]. The final incubation volume (100 µL) contained 1 µg of protein and the substrate concentration was 20 µM (10 nM of ^3^H-anandamide having specific activity of 60 Ci/mmol and concentration of 1 mCi/mL).

### Reversibility testing

Reversibility testing was carried out as previously described [31]. Briefly, DMSO/inhibitor (0.5 µl) was incubated together with hABHD12-HEK293 lysate (4.5 µl, 0.3 µg protein/well) in TEMN-BSA buffer for 30 min. After this, a 40-fold dilution of enzyme-inhibitor complex was brought about by addition of the substrate [1-AG, 12.5 µM final concentration, containing additionally 1% EtOH (v/v)] in 195 µl of glycerol assay mix and fluorescence was kinetically monitored at 10 min intervals for 90 min at RT. Five to six inhibitor concentrations were included to cover the entire dose-response range and the IC_50_ values were determined at time-points 10, 20, 30, 60 and 90 min.

### Determination of ABHD12 activity using a LC-MS assay

Betulinic acid, oleanolic acid, ursolic acid as well as the compounds **18** and **25** at concentrations >10^−5^ M were found to interfere with the routine fluorescent assay, and therefore, the dose-response curves for these compounds were determined using LC-MS. The fluorescent glycerol assay was mimicked otherwise but instead of assaying glycerol, utilization of 1-AG and concomitant formation of AA was monitored by LC-MS analysis. Briefly, after 30 minutes preincubation of DMSO/inhibitor (1 µl) with hABHD12-HEK293 cell lysate (99 µl, 0.3 µg protein/well) in TEMN-BSA buffer, pH 7.4, 1-AG [12.5 µM final concentration, containing additionally 1% EtOH (v/v)] in TEMN-BSA buffer (100 µl) was added and the incubation continued for 90 min at RT. Enzymatic reaction was stopped by adding 400 µL of cold 11 mM H_3_PO_4_ in acetonitrile. 150 µL aliquots were filtered using Captiva 96-well filter plate (0.2 µm, Agilent Technologies, USA) into the Captiva 96-well collection plate (Agilent Technologies, USA) containing 50 µL of 0.1% formic acid in H_2_O. The collection plate was centrifuged at 4°C for 10 minutes and the samples were analyzed by LC-MS. HPLC system consisted of an Agilent 1200 Series Rapid Resolution LC System (Agilent Technologies, Waldbronn, Germany) with a solvent micro vacuum degasser, a binary pump, a thermostatted column compartment, and an autosampler. The mass analysis was made with an Agilent 6410 Triple Quadrupole MS equipped with an electrospray ionization (ESI) source (Agilent Technologies, Palo Alto, CA, USA). Five microliters of the sample solution were injected onto a reversed-phase HPLC column (Zorbax Eclipse XDB-C18 Rapid Resolution HT 2.1 mm×50 mm, 1.8 µm) (Agilent Technologies, Palo Alto, CA, USA) using an isocratic mobile phase consisting of 0.1% formic acid in a solution of H_2_O and methanol (20∶80 v/v) delivered at 0.5 mL/min. Column temperature was maintained at 50°C and the autosampler tray temperature was set at 4°C. The following ionization conditions were used for 1-AG: positive ESI mode, drying gas (nitrogen) temperature 300°C, drying gas flow rate 8 L/min, nebulizer pressure 40 psi, and capillary voltage 4000 V. The following ionization conditions were used for AA: negative ESI mode, drying gas (nitrogen) temperature 300°C, drying gas flow rate 8 L/min, nebulizer pressure 40 psi, and capillary voltage 4000 V. Analyte detection was performed using multiple reaction monitoring (MRM) with the following transitions: *m/z* 379 → 287 for 1-AG and *m/z* 303 → 303 for AA. Fragmentor voltage and collision energy for 1-AG and AA were 130 V/8 V and 175 V/2 V, respectively. Retention times for 1-AG and AA were 2.4 and 3.4 min, respectively. In addition, the 2-AG positional isomer is chromatographically separated from 1-AG with retention time 2.1 min, and the peak areas of 1-AG and 2-AG were subsequently combined for all quantitative analyses reported and are thus collectively termed “1-AG”. The calibration range of 0.1–2,000 ng/mL and 0.1–1,000 ng/mL was prepared for monitored 1-AG and AA, respectively. The lower limit of quantification (LLOQ) for both analytes, 1-AG and AA, was 1.0 ng/mL.

### Cannabinoid receptor activity

The CB_1_ and CB_2_ receptor activity of the selected compounds (10 µM) were measured using the GTPγS binding assay according to the previously described methods [32], [33].

### Activity-based protein profiling (ABPP) of serine hydrolases

Competitive ABPP using HEK293 cell lysates and mouse brain membranes was conducted to visualize effects of selected triterpenoids on the binding of the active site serine-targeting fluorescent fluorophosphonate probe TAMRA-FP following outlines of previous protocols [4], [31]. Briefly, lysates (25 µg) of HEK293 cells with or without (Mock) hABHD12 overexpression or mouse wholebrain membranes (100 µg) were treated for 1 h with DMSO or the inhibitor, after which TAMRA-FP labelling was conducted for 5 min (lysates) or 1 hour (membranes) at RT (final probe concentration 2 µM). The reaction was quenched by addition of gel loading buffer (followed by boiling for 5 min in the case of lysates), after which 10 µg protein (10 µl) was loaded per lane and the proteins were resolved in 10% SDS-PAGE together with standards. TAMRA-FP labeling was visualized (λ_ex_ 552; λ_em_ 575 nm) by a fluorescent reader (FLA-3000 laser fluorescence scanner, Fujifilm, Tokyo, Japan). The intensity of bands was quantified using ImageJ, a freely available image analysis software (http://rsbweb.nih.gov/ij/).

### Molecular modelling

Structures of small molecules were prepared using the LigPrep module Schrödinger's Maestro software package (Schrödinger Release 2013-2: Maestro version 9.5, LigPrep version 2.7, MacroModel, version 10.1, Schrödinger, LLC, New York, NY). Deprotonated forms of the carboxylic acids were used because that would be the predominant form of such structures at the physiological pH. The conformational database was constructed using the conformational search with default torsional sampling settings (MCMM) embedded to Macromodel module of Maestro. The full dataset of 42 triterpenoids studied here resulted in a database of 1703 conformations. The pharmacophore model was constructed using Molecular Operating Environment (MOE) software, version 2013.08 (Chemical Computing Group Inc., Canada). Active structures were overlaid using flexible alignment and the pharmacophoric elements were defined using compounds **23** and **33** as main templates. Nine hydrophobic features were used to describe the planar shape of the lupane skeleton. Three hydrogen bond acceptor features were defined using the carboxyl group at position 17, which was found to provide a good match for all active structures including also groups such as oxime and aldehyde substructures. In the case of substituents in the position 3 of the lupane skeleton both hydrogen bond donor and accepting groups can be found. We used compound **33** for placement of the hydrogen bond donating site and compound **23** for adding the hydrogen bond acceptor site. An additional acceptor site was needed for covering compounds having hydroxyl group in the position 3. Finally, a ligand shape constraint was defined using the compound **34** as template in order to rule out bigger inactive structures such as esters in the position 17. In the final model, two partial hit constraints (at least one) were added for the groups of features describing hydrogen bond interactions at the positions 3 and 17.

### Data analysis

The inhibitor dose-response curves and IC_50_ values derived thereof were calculated from nonlinear regressions using GraphPad Prism 5.0 for Windows (GraphPad Software, San Diego California USA, www.graphpad.com) and the results are presented as mean ± S.E.M. of at least three independent experiments performed in duplicate. Statistical comparisons between hABHD12 band intensities in the ABPP study (Figure 6B) and between the IC_50_ values in the reversibility study (Figure S4) were tested using one-way ANOVA, followed by Tukey's multiple comparison test with *p*<0.05 considered as statistically significant (*P<0.05, **P<0.01, and ***P<0.001).

### Ethics statement

For ABPP experiments (Figure 6C) and cannabinoid receptor activity assays (Table S2), membranes prepared from brain tissue of 4-week-old male rodents were used. The animals were obtained from the National Laboratory Animal Centre, University of Eastern Finland. Approval for the harvesting of animal tissue was obtained from the local welfare officer of the University of Eastern Finland. No further ethical approval was required, as the experiments did not involve any *in vivo* treatment.

## Supporting Information

Figure S1 Established natural substrates (LPS, lysophosphatidylserine; 1(3)-AG, 1(3)-arachidonoyl glycerol) and inhibitors of ABHD12 (THL, tetrahydrolipstatin; MAFP, methyl arachidonyl fluorophosphonate), as well as general structure and numbering system of the lupane skeleton.
(PDF)

Figure S2 Chemical structures of commercial compounds **1–15**.
(PDF)

Figure S3 Hydrogen bond donor or acceptor at the position 3 is one of the key determinants for the inhibitory activity of betulinic acid derivatives towards hABHD12.
(PDF)

Figure S4 Reversibility of hABHD12 inhibition by the triterpenoids. Fast 40-fold dilution of inhibitor-treated hABHD12-HEK293 lysate preparation (see experimental procedures for further details) results in time-dependent drop of inhibitor potency, as evidenced for the triterpenoids **8** (maslinic acid), **33** and **34**. In contrast, the potency for the established irreversible serine hydrolase inhibitor THL (orlistat) does not change in a statistically significant manner during the time-course of this study. Due to low signal-to-noise ratio, no reliable data could be obtained for compound **34** and THL at time-point 10 min; therefore these data points are not presented. Data are mean ± SEM from three independent experiments. Statistical differences between IC_50_ values at the earliest (10 or 20 min) and other time-points were tested using one-way ANOVA, followed by Tukey's multiple comparison test (*P<0.05, **P<0.01 and ***P<0.001).
(PDF)

Figure S5 Lysophosphatidylserine (LPS, orange carbons, panel **A**), the *in vivo* substrate of ABHD12, and tetrahydrolipstatin (THL, green carbons, panel **B**), the irreversible inhibitor of ABHD12, superimposed with the reversible inhibitor betulinic acid (gray carbons). LPS and THL were modeled in an extended conformation and the hydrocarbon chains have been partly faded out. Note that even though the overall shapes of the molecules are quite different, alignment shows that the topological distance and orientation of the important functional moieties is surprisingly similar. Namely, carboxylic acid group of LPS and formyl group of THL both align with the carboxyl group of betulinic acid (dark gray circles). In addition, hydroxyl group of betulinic acid aligns with ester carbonyl of LPS and lactone carbonyl of THL (light gray circles).
(PDF)

Table S1 Inhibitory activity of selected triterpenoids against hABHD6, hMAGL and hFAAH.
(PDF)

Table S2 Activity of selected compounds at CB_1_ and CB_2_ receptors. Compounds were tested at 10 µM concentration.
(PDF)

Table S3 Chemical structures of tested compounds **44–67** that do not markedly inhibit hABHD12 when tested at 10 µM concentration.
(PDF)

## References

[1] Blankman JL, Simon GM, Cravatt BF (2007) A comprehensive profile of brain enzymes that hydrolyze the endocannabinoid 2-arachidonoylglycerol. Chem Biol 14: 1347–1356. 18096503 10.1016/j.chembiol.2007.11.006 PMC2692834

[2] Blankman JL, Long JZ, Trauger SA, Siuzdak G, Cravatt BF (2013) ABHD12 controls brain lysophosphatidylserine pathways that are deregulated in a murine model of the neurodegenerative disease PHARC. Proc Natl Acad Sci U S A 110: 1500–1505. 23297193 10.1073/pnas.1217121110 PMC3557017

[3] Fiskerstrand T, H'Mida-Ben Brahim D, Johansson S, M'Zahem A, Haukanes BI, et al (2010) Mutations in ABHD12 cause the neurodegenerative disease PHARC: An inborn error of endocannabinoid metabolism. Am J Hum Genet 87: 410–417. 20797687 10.1016/j.ajhg.2010.08.002 PMC2933347

[4] Navia-Paldanius D, Savinainen JR, Laitinen JT (2012) Biochemical and pharmacological characterization of human alpha/beta-hydrolase domain containing 6 (ABHD6) and 12 (ABHD12). J Lipid Res 53: 2413–2424. 22969151 10.1194/jlr.M030411 PMC3466009

[5] Jager S, Trojan H, Kopp T, Laszczyk MN, Scheffler A (2009) Pentacyclic triterpene distribution in various plants - rich sources for a new group of multi-potent plant extracts. Molecules 14: 2016–2031. 19513002 10.3390/molecules14062016 PMC6254168

[6] Moura-Letts G, Villegas LF, Marcalo A, Vaisberg AJ, Hammond GB (2006) In vivo wound-healing activity of oleanolic acid derived from the acid hydrolysis of *Anredera diffusa* . J Nat Prod 69: 978–979. 16792424 10.1021/np0601152

[7] He X, Liu RH (2007) Triterpenoids isolated from apple peels have potent antiproliferative activity and may be partially responsible for apple's anticancer activity. J Agric Food Chem 55: 4366–4370. 17488026 10.1021/jf063563o

[8] Li L, Zhang X, Cui L, Wang L, Liu H, et al (2013) Ursolic acid promotes the neuroprotection by activating Nrf2 pathway after cerebral ischemia in mice. Brain Res 1497: 32–39. 23276496 10.1016/j.brainres.2012.12.032

[9] Siewert B, Wiemann J, Köwitsch A, Csuk R (2013) The chemical and biological potential of C ring modified triterpenoids. Eur J Med Chem 72: 84–101. 24361521 10.1016/j.ejmech.2013.11.025

[10] Alakurtti S, Makela T, Koskimies S, Yli-Kauhaluoma J (2006) Pharmacological properties of the ubiquitous natural product betulin. Eur J Pharm Sci 29: 1–13. 16716572 10.1016/j.ejps.2006.04.006

[11] Castellano JM, Guinda A, Delgado T, Rada M, Cayuela JA (2013) Biochemical basis of the antidiabetic activity of oleanolic acid and related pentacyclic triterpenes. Diabetes 62: 1791–1799. 23704520 10.2337/db12-1215 PMC3661625

[12] Fulda S (2008) Betulinic acid for cancer treatment and prevention. Int J Mol Sci 9: 1096–107. 19325847 10.3390/ijms9061096 PMC2658785

[13] Pollier J, Goossens A (2012) Oleanolic acid. Phytochemistry 77: 10–15. 22377690 10.1016/j.phytochem.2011.12.022

[14] Yadav VR, Prasad S, Sung B, Kannappan R, Aggarwal BB (2010) Targeting inflammatory pathways by triterpenoids for prevention and treatment of cancer. Toxins (Basel) 2: 2428–2466. 22069560 10.3390/toxins2102428 PMC3153165

[15] Safe SH, Prather PL, Brents LK, Chadalapaka G, Jutooru I (2012) Unifying mechanisms of action of the anticancer activities of triterpenoids and synthetic analogs. Anticancer Agents Med Chem 12: 1211–1220. 22583404 10.2174/187152012803833099 PMC3532564

[16] Salminen A, Lehtonen M, Suuronen T, Kaarniranta K, Huuskonen J (2008) Terpenoids: natural inhibitors of NF-kappaB signaling with anti-inflammatory and anticancer potential. Cell Mol Life Sci 65: 2979–2999. 18516495 10.1007/s00018-008-8103-5 PMC11131807

[17] King AR, Dotsey EY, Lodola A, Jung KM, Ghomian A, et al (2009) Discovery of potent and reversible monoacylglycerol lipase inhibitors. Chem Biol 16: 1045–1052. 19875078 10.1016/j.chembiol.2009.09.012 PMC3034734

[18] Laitinen T, Navia-Paldanius D, Rytilahti R, Marjamaa J, Kařízková J, et al (2014) Mutation of Cys242 of human monoacylglycerol lipase disrupts balanced hydrolysis of 1- and 2-monoacylglycerols and selectively impairs inhibitor potency. Mol Pharmacol 85: 510–519. 24368842 10.1124/mol.113.090795

[19] da Silva K, Paszcuk A, Passos G, Silva E, Bento A, et al (2011) Activation of cannabinoid receptors by the pentacyclic triterpene α,β-amyrin inhibits inflammatory and neuropathic persistent pain in mice. Pain 152: 1872–1887. 21620566 10.1016/j.pain.2011.04.005

[20] Chicca A, Marazzi J, Gertsch J (2012) The antinociceptive triterpene beta-amyrin inhibits 2-arachidonoylglycerol (2-AG) hydrolysis without directly targeting cannabinoid receptors. Br J Pharmacol 167: 1596–1608. 22646533 10.1111/j.1476-5381.2012.02059.x PMC3525863

[21] Genet C, Strehle A, Schmidt C, Boudjelal G, Lobstein A, et al (2010) Structure-activity relationship study of betulinic acid, a novel and selective TGR5 agonist, and its synthetic derivatives: potential impact in diabetes. J Med Chem 53: 178–190. 19911773 10.1021/jm900872z

[22] Liu Q, Liu H, Zhang L, Guo T, Wang P, et al (2013) Synthesis and antitumor activities of naturally occurring oleanolic acid triterpenoid saponins and their derivatives. Eur J Med Chem 64: 1–15. 23639650 10.1016/j.ejmech.2013.04.016

[23] Gao H, Wu L, Kuroyanagi M, Harada K, Kawahara N, et al (2003) Antitumor-promoting constituents from *Chaenomeles sinensis* KOEHNE and their activities in JB6 mouse epidermal cells. Chem Pharm Bull (Tokyo) 51: 1318–1321. 14600382 10.1248/cpb.51.1318

[24] Alakurtti S, Heiska T, Kiriazis A, Sacerdoti-Sierra N, Jaffe CL, et al (2010) Synthesis and anti-leishmanial activity of heterocyclic betulin derivatives. Bioorg Med Chem 18: 1573–1582. 20116263 10.1016/j.bmc.2010.01.003

[25] Pohjala L, Alakurtti S, Ahola T, Yli-Kauhaluoma J, Tammela P (2009) Betulin-derived compounds as inhibitors of alphavirus replication. J Nat Prod 72: 1917–1926. 19839605 10.1021/np9003245

[26] Kumar NS, Muthukuda PM, Wazeer MIM (1985) A lupenediol from *Euonymus revolutus* . Phytochemistry 24: 1337–1340.

[27] Haavikko R, Nasereddin A, Sacerdoti-Sierra N, Kopelyanskiy D, Alakurtti S, et al (2014) Heterocycle-fused lupane triterpenoids inhibit *Leishmania donovani* amastigotes. Med Chem Commun 5: 445–451.

[28] Hao J, Sun H, Zhang P, Zhang X, Liu J, et al (2009) Efficient access to isomeric 2,3-dihydroxy lupanes: first synthesis of alphitolic acid. Tetrahedron 65: 7975–7984.

[29] Santos RC, Salvador JA, Marin S, Cascante M, Moreira JN, et al (2010) Synthesis and structure-activity relationship study of novel cytotoxic carbamate and N-acylheterocyclic bearing derivatives of betulin and betulinic acid. Bioorg Med Chem 18: 4385–4396. 20494586 10.1016/j.bmc.2010.04.085

[30] Saario SM, Poso A, Juvonen RO, Jarvinen T, Salo-Ahen OM (2006) Fatty acid amide hydrolase inhibitors from virtual screening of the endocannabinoid system. J Med Chem 49: 4650–4656. 16854070 10.1021/jm060394q

[31] Aaltonen N, Savinainen JR, Ribas CR, Ronkko J, Kuusisto A, et al (2013) Piperazine and piperidine triazole ureas as ultrapotent and highly selective inhibitors of monoacylglycerol lipase. Chem Biol 20: 379–390. 23521796 10.1016/j.chembiol.2013.01.012

[32] Savinainen JR, Kokkola T, Salo OM, Poso A, Jarvinen T, et al (2005) Identification of WIN55212-3 as a competitive neutral antagonist of the human cannabinoid CB2 receptor. Br J Pharmacol 145: 636–645. 15852035 10.1038/sj.bjp.0706230 PMC1576178

[33] Savinainen JR, Saario SM, Niemi R, Jarvinen T, Laitinen JT (2003) An optimized approach to study endocannabinoid signaling: evidence against constitutive activity of rat brain adenosine A1 and cannabinoid CB1 receptors. Br J Pharmacol 140: 1451–1459. 14623770 10.1038/sj.bjp.0705577 PMC1574161

[34] Hoover HS, Blankman JL, Niessen S, Cravatt BF (2008) Selectivity of inhibitors of endocannabinoid biosynthesis evaluated by activity-based protein profiling. Bioorg Med Chem Lett 18: 5838–5841. 18657971 10.1016/j.bmcl.2008.06.091 PMC2634297

[35] Patel JZ, Parkkari T, Laitinen T, Kaczor AA, Saario SM, et al (2013) Chiral 1,3,4-oxadiazol-2-ones as highly selective FAAH inhibitors. J Med Chem 56: 8484–8496. 24083878 10.1021/jm400923s

