# Supplementary figures and images for: Discovery of Triterpenoids as Reversible Inhibitors of α/β-hydrolase Domain Containing 12 (ABHD12)
Source: PLoS One. 2014 May 30;9(5):e98286. doi: 10.1371/journal.pone.0098286 (PMC4045134; doi:10.1371/journal.pone.0098286)

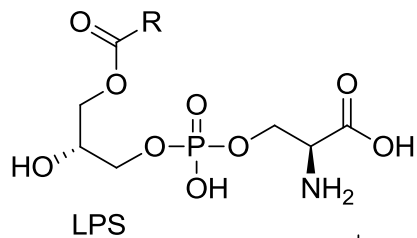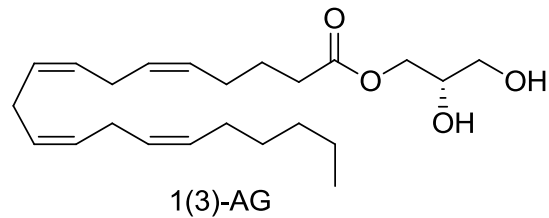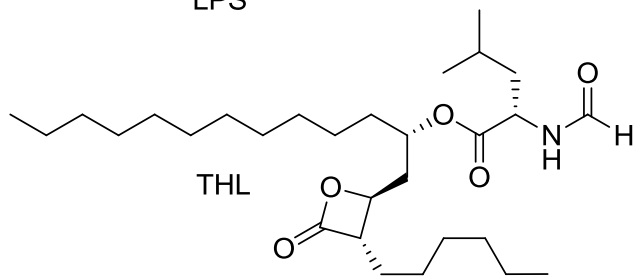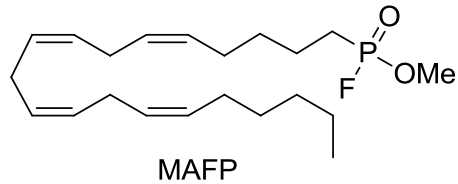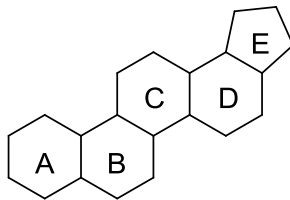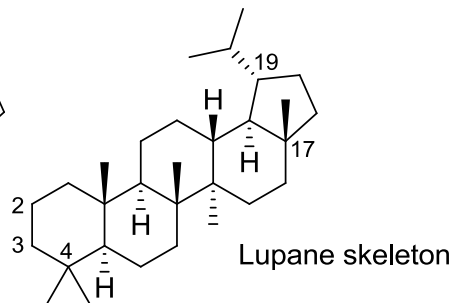

Supplement: Figure S1 — Established natural substrates (LPS, lysophosphatidylserine; 1(3)-AG, 1(3)-arachidonoyl glycerol) and inhibitors of ABHD12 (THL, tetrahydrolipstatin; MAFP, methyl arachidonyl fluorophosphonate), as well as general structure and numbering system of the lupane skeleton. (PDF) [file pone.0098286.s001.pdf]

### Lupanes

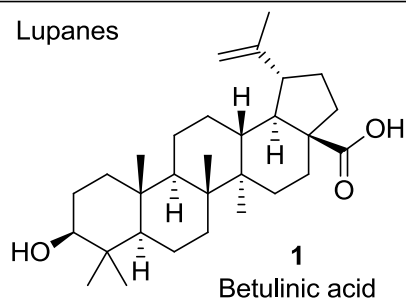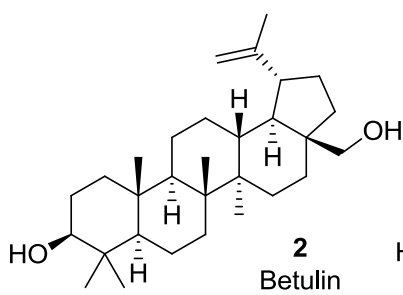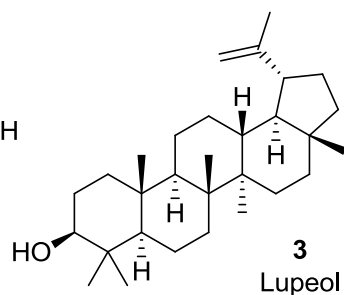

### Ursanes

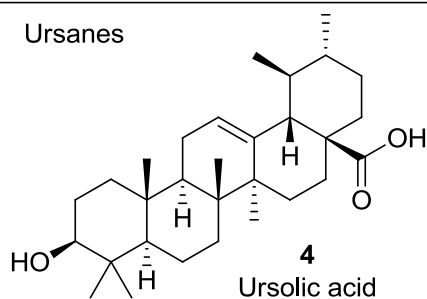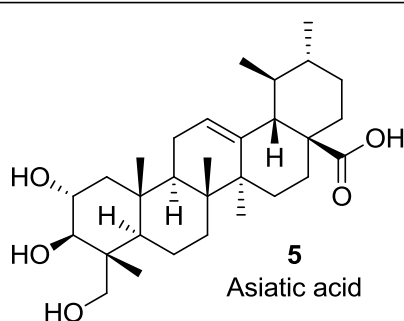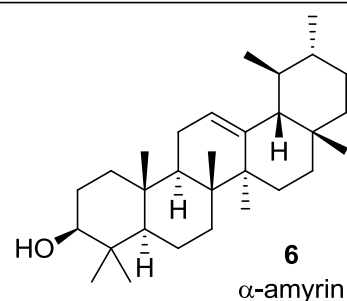

### Oleananes

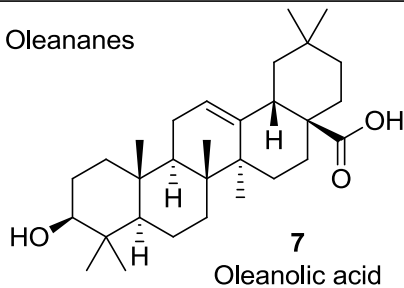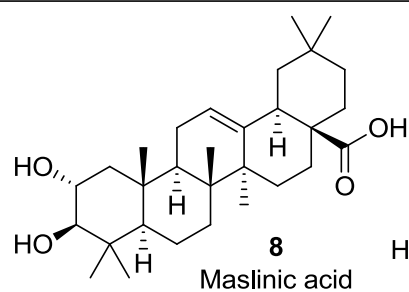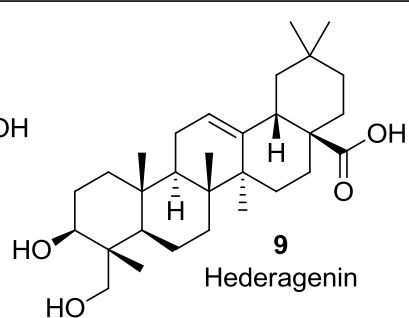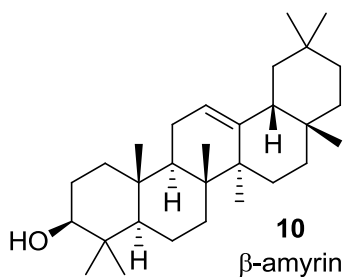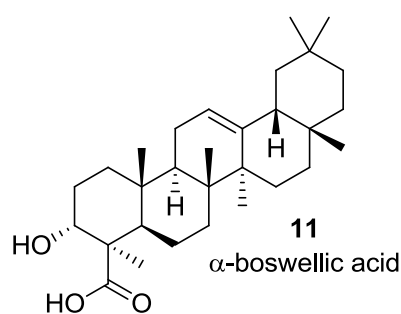

### Triterpenoids

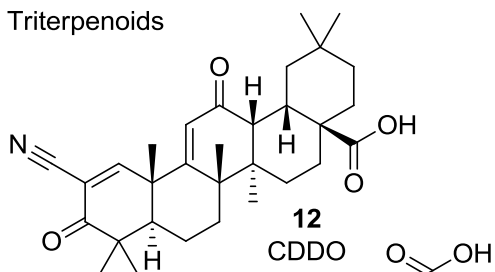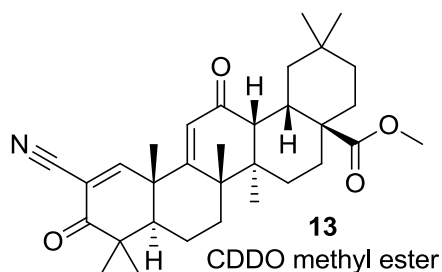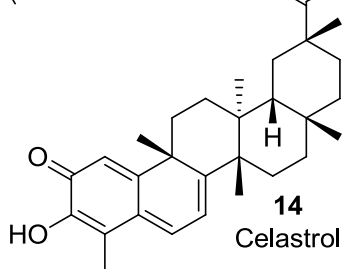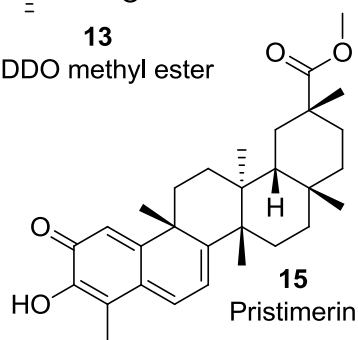

Supplement: Figure S2 — Chemical structures of commercial compounds 1–15. (PDF) [file pone.0098286.s002.pdf]

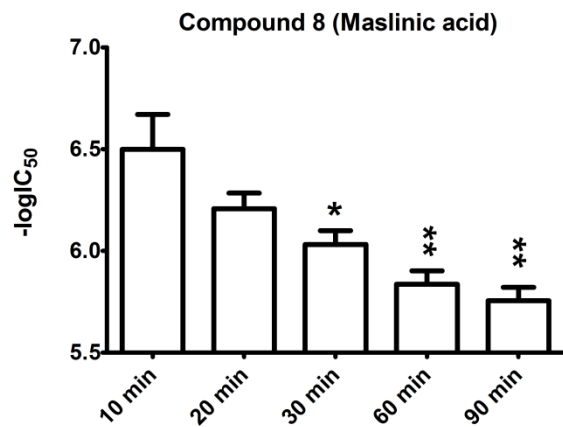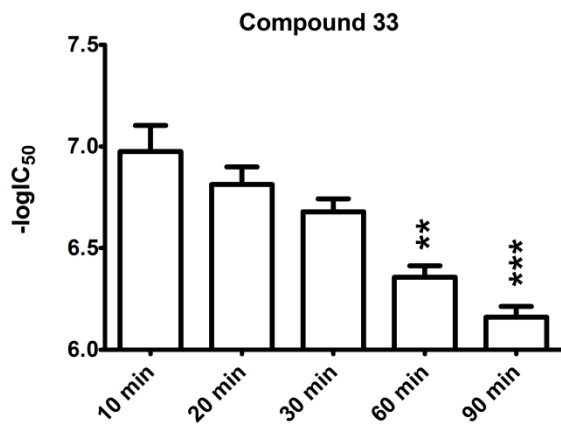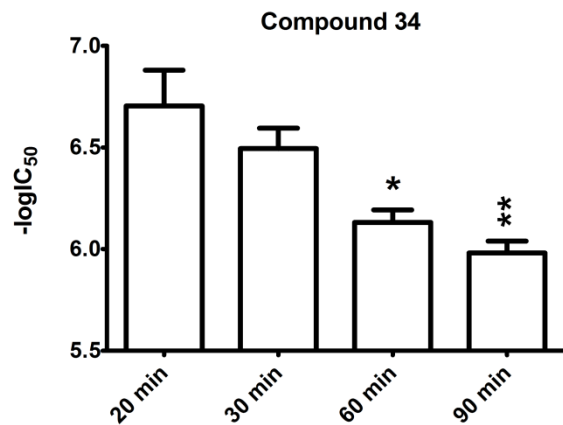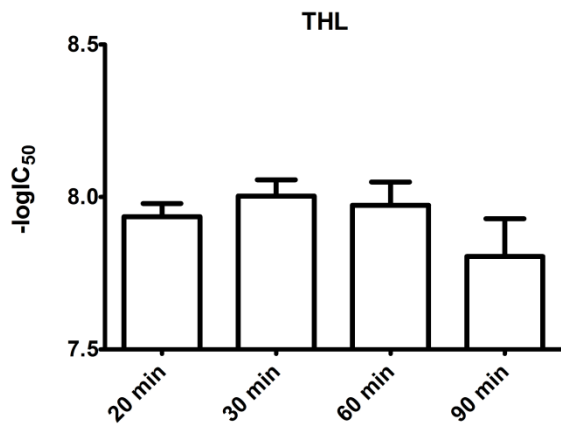

Supplement: Figure S4 — Reversibility of hABHD12 inhibition by the triterpenoids. Fast 40-fold dilution of inhibitor-treated hABHD12-HEK293 lysate preparation (see experimental procedures for further details) results in time-dependent drop of inhibitor potency, as evidenced for the triterpenoids 8 (maslinic acid), 33 and 34. In contrast, the potency for the established irreversible serine hydrolase inhibitor THL (orlistat) does not change in a statistically significant manner during the time-course of this study. Due to low signal-to-noise ratio, no reliable data could be obtained for compound 34 and THL at time-point 10 min; therefore these data points are not presented. Data are mean ± SEM from three independent experiments. Statistical differences between IC50 values at the earliest (10 or 20 min) and other time-points were tested using one-way ANOVA, followed by Tukey's multiple comparison test (*P<0.05, **P<0.01 and ***P<0.001). (PDF) [file pone.0098286.s004.pdf]

**A** Betulinic acid - **LPS**

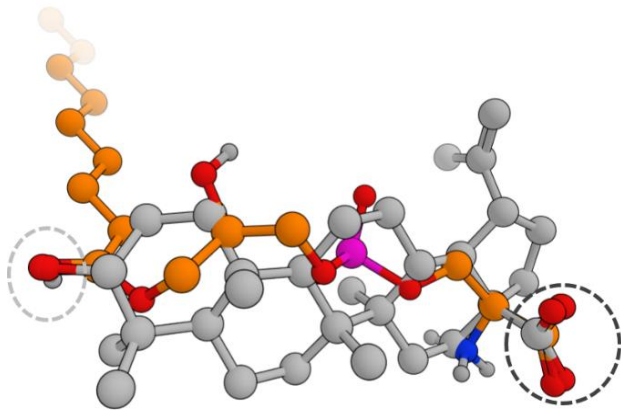

**B** Betulinic acid - **THL**

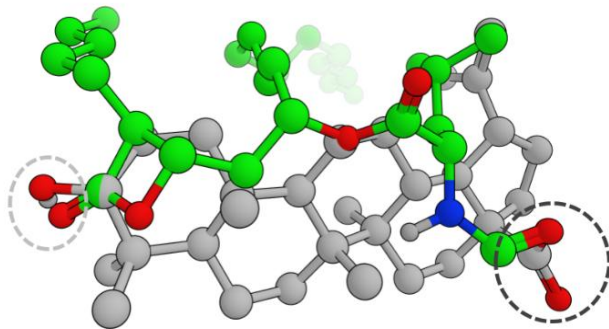

Supplement: Figure S5 — Lysophosphatidylserine (LPS, orange carbons, panel A), the in vivo substrate of ABHD12, and tetrahydrolipstatin (THL, green carbons, panel B), the irreversible inhibitor of ABHD12, superimposed with the reversible inhibitor betulinic acid (gray carbons). LPS and THL were modeled in an extended conformation and the hydrocarbon chains have been partly faded out. Note that even though the overall shapes of the molecules are quite different, alignment shows that the topological distance and orientation of the important functional moieties is surprisingly similar. Namely, carboxylic acid group of LPS and formyl group of THL both align with the carboxyl group of betulinic acid (dark gray circles). In addition, hydroxyl group of betulinic acid aligns with ester carbonyl of LPS and lactone carbonyl of THL (light gray circles). (PDF) [file pone.0098286.s005.pdf]
