# Supplementary material for: Discovery of Triterpenoids as Reversible Inhibitors of α/β-hydrolase Domain Containing 12 (ABHD12)
Source: PLoS One. 2014 May 30;9(5):e98286. doi: 10.1371/journal.pone.0098286 (PMC4045134; doi:10.1371/journal.pone.0098286)
Supplement: Figure S3 — Hydrogen bond donor or acceptor at the position 3 is one of the key determinants for the inhibitory activity of betulinic acid derivatives towards hABHD12. (PDF) [file pone.0098286.s003.pdf]

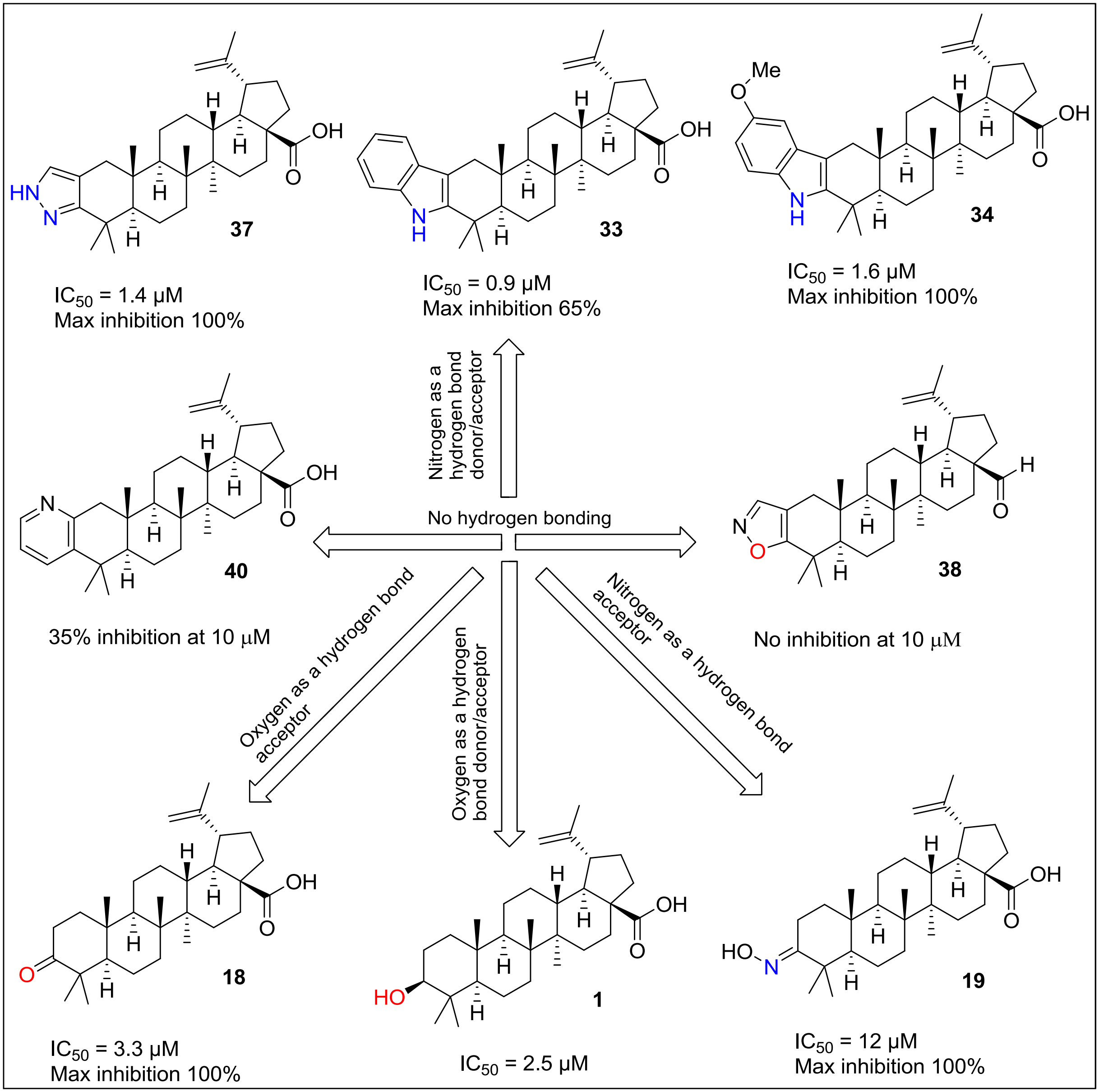

37
IC50 = 1.4 μM
Max inhibition 100%
33
IC50 = 0.9 μM
Max inhibition 65%
34
IC50 = 1.6 μM
Max inhibition 100%
Nitrogen as a hydrogen bond donor/acceptor
40
35% inhibition at 10 μM
No hydrogen bonding
38
No inhibition at 10 μM
Oxygen as a hydrogen bond acceptor
Oxygen as a hydrogen bond donor/acceptor
Nitrogen as a hydrogen bond acceptor
18
IC50 = 3.3 μM
Max inhibition 100%
1
IC50 = 2.5 μM
19
IC50 = 12 μM
Max inhibition 100%
