# Supplementary material for: Discovery of Triterpenoids as Reversible Inhibitors of α/β-hydrolase Domain Containing 12 (ABHD12)
Source: PLoS One. 2014 May 30;9(5):e98286. doi: 10.1371/journal.pone.0098286 (PMC4045134; doi:10.1371/journal.pone.0098286)
Supplement: Table S1 — Inhibitory activity of selected triterpenoids against hABHD6, hMAGL and hFAAH. (PDF) [file pone.0098286.s006.pdf]

Compound (10 µM)	hABHD6	hMAGL	hFAAH
	Remaining activity % control ± s.e.m (n=3)		Remaining activity % control (mean (range)), n=2
**1** (Betulinic acid)	~ 70	76.7 ± 0.1	83 (80-85)
**8** (Maslinic acid)	NI	95.6 ± 0.8	83 (82-84)
**17**	93.6 ± 3.8	84.1 ± 4.8	95 (88-101)
**19**	85.8 ± 3.0	86.6 ± 2.0	91 (91-91)
**23**	83.0 ± 2.8	77.5 ± 3.1	98 (92-104)
**24**	92.4 ± 3.9	86.0 ± 4.3	95 (88-101)
**25**	103.2 ± 2.3	88.7 ± 4.2	97 (90-104)
**31**	93.2 ± 3.0	82.7 ± 4.1	91 (87-95)
**33**	81.0 ± 4.3	74.8 ± 4.6	90 (85-95)
**36**	80.0 ± 4.4	54.1 ± 4.7	88 (88-88)
**41**	85.4 ± 3.6	79.4 ± 4.1	90 (86-93)
**42**	88.7 ± 4.3	79.8 ± 4.1	93 (91-95)

NI, no inhibition at 10 µM concentration
