# Supplementary material for: Discovery of Triterpenoids as Reversible Inhibitors of α/β-hydrolase Domain Containing 12 (ABHD12)
Source: PLoS One. 2014 May 30;9(5):e98286. doi: 10.1371/journal.pone.0098286 (PMC4045134; doi:10.1371/journal.pone.0098286)
Supplement: Table S2 — Activity of selected compounds at CB1 and CB2 receptors. Compounds were tested at 10 µM concentration. (PDF) [file pone.0098286.s007.pdf]

Compound	CB_1R agonist activity (rat cerebellar membranes)	CB_2R agonist activity (hCB_2R-CHO cell membranes)	CB_1R antagonist activity (rat cerebellar membranes)
	[^{35}S]GTPγS binding % Basal (Mean (range), n=2)		[^{35}S]GTPγS binding % HU210-evoked response (Mean (range), n=2)
**1** (Betulinic acid)	91 (87-96)	132 (106-158)	99 (98-99)
**8** (Maslinic acid)	93 (88-97)	114 (104-123)	99 (97-102)
**23**	89 (86-92)	98 (95-100)	92 (90-95)
**33**	95 (94-96)	102 (90-114)	97 (96-98)
HU210 (1 µM)	430 ± 36[1]	-	-
HU210 (10 nM)	-	354 (348-361)	-
AM251 (1 µM)	88 ± 1.5[1]	-	42 ± 2.7[1]
SR144528 (1 µM)	-	68 (60-76)	-

[1] Results are % basal ± SEM, n > 3
