# Supplementary material for: Discovery of Triterpenoids as Reversible Inhibitors of α/β-hydrolase Domain Containing 12 (ABHD12)
Source: PLoS One. 2014 May 30;9(5):e98286. doi: 10.1371/journal.pone.0098286 (PMC4045134; doi:10.1371/journal.pone.0098286)
Supplement: Table S3 — Chemical structures of tested compounds 44–67 that do not markedly inhibit hABHD12 when tested at 10 µM concentration. (PDF) [file pone.0098286.s008.pdf]

**Compound**	**Structure**	Remaining activity % control ± s.e.m (n=3)
**44**	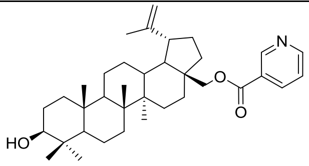	95 ± 0.3
**45**	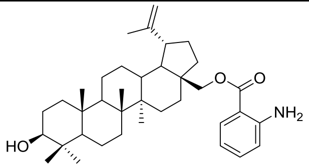	99 ± 0.6
**46**	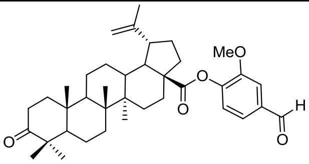	92 ± 0.0
**47**	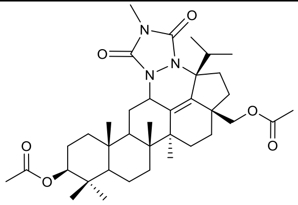	95 ± 0.3
**48**	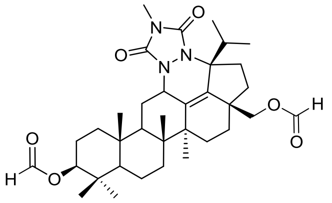	85 ± 0.6
**49**	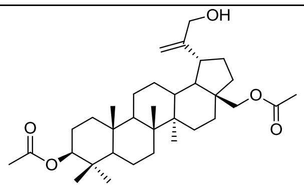	95 ± 0.3
**50**	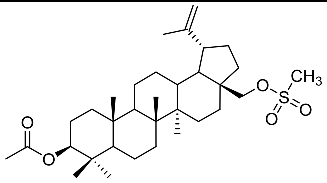	94 ± 0.9

**51**	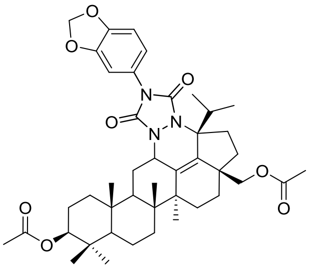	100 ± 3.3
**52**	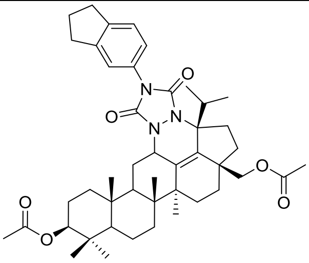	93 ± 0.9
**53**	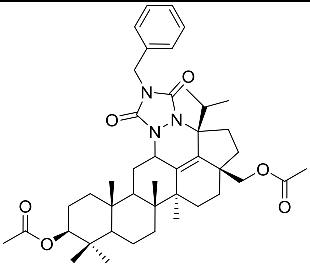	82 ± 0.7
**54**	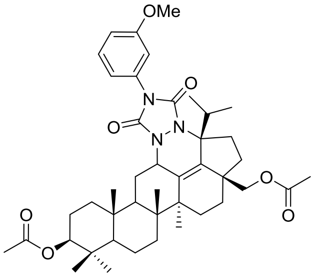	97 ± 0.5
**55**	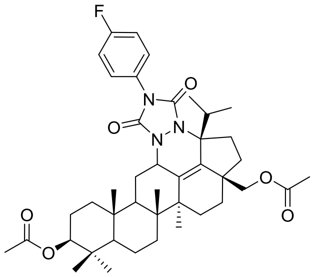	90 ± 0.4
**56**	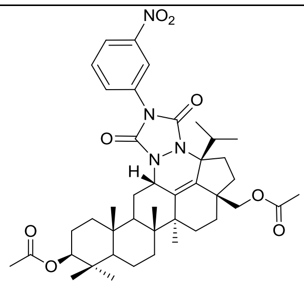	92 ± 0.5

**57**	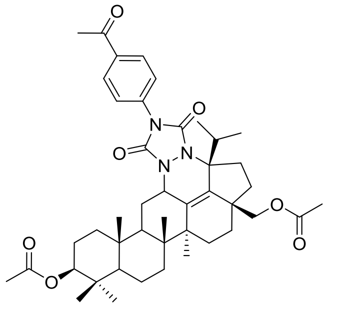	97 ± 1.0
**58**	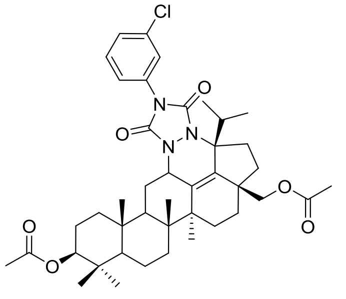	88 ± 1.3
**59**	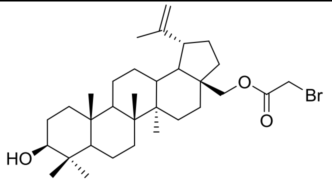	86 ± 1.5
**60**	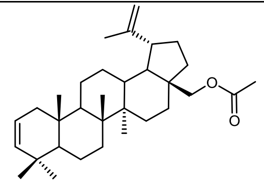	93 ± 0.4
**61**	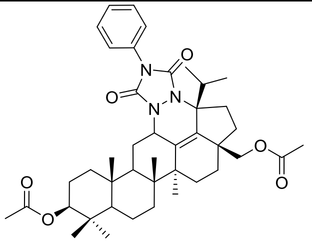	96 ± 1.0
**62**	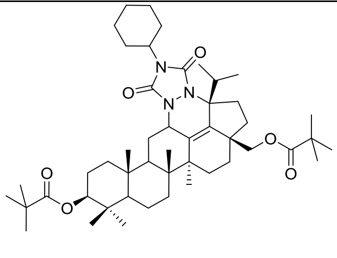	98 ± 0.2

**63**	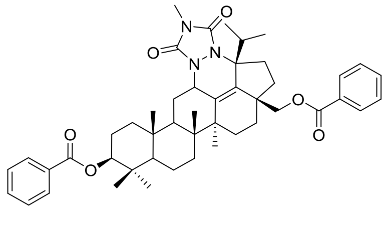	93 ± 1.7
**64**	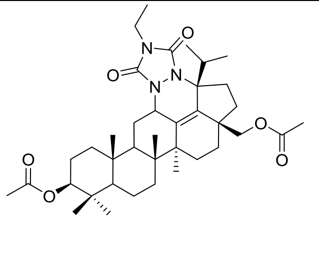	93 ± 0.2
**65**	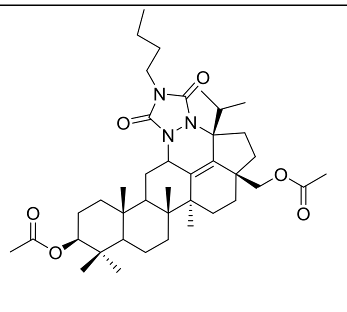	91 ± 0.6
**66**	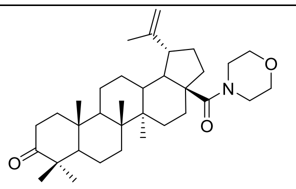	77 ± 2.0
**67**	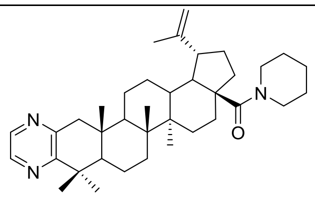	91 ± 0.6
**68**	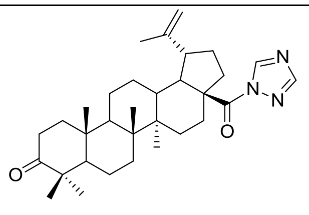	77 ± 1.1
